# Bacterial Cellulose- and Laponite-Reinforced Corn Starch Bioplastics for Sustainable Packaging

**DOI:** 10.3390/polym18182190

**Published:** 2026-09-08

**Authors:** Rysgul Tuleyeva, Nargiz Gizatullina, Alexey Shakhvorostov, Zhanserik Shynykul, Gaukhar Toleutay

**Affiliations:** 1 Department of Chemical and Biochemical Engineering, Geology and Oil-Gas Business Institute Named After K. Turyssov, Satbayev University, Almaty 050043, Kazakhstan; rysgultuleyeva@gmail.com (R.T.); gizatullinanargiz.nz@gmail.com (N.G.); 2 Department of Chemistry and Technology of Organic Substances, Natural Compounds and Polymers, Faculty of Chemistry and Chemical Technology, Al-Farabi Kazakh National University, Almaty 050040, Kazakhstan; 3 Institute of Polymer Materials and Technology, Almaty 050019, Kazakhstan; a.shakhvorostov@ipmt.kz; 4 Department of Pharmaceutical Sciences, College of Pharmacy, The University of Tennessee, Health Science Center, Memphis, TN 38103, USA; 5 Department of Chemistry, University of Tennessee, Knoxville, TN 37996, USA

**Keywords:** corn starch, bacterial cellulose, carboxylated cellulose nanofibers, α-cellulose, laponite

## Abstract

Growing environmental concerns associated with petroleum-based plastics have stimulated the development of renewable and biodegradable alternatives. In this study, corn-starch-based composite films were prepared with bacterial cellulose (BC), α-cellulose (α-C), or carboxylated cellulose nanofibers (CNC) in the presence of laponite and glycerol. The films were characterized using Fourier-transform infrared (FTIR) spectroscopy, thermogravimetric analysis, optical measurements at 600 nm, tensile testing, qualitative solvent-exposure tests, and thermally induced repair experiments. Among the films containing different cellulose types, the bacterial-cellulose-containing bioplastic (BC-BP) exhibited the highest tensile strength and Young’s modulus, reaching 4.47 and 0.229 MPa, respectively. The carboxylated-cellulose-nanofiber-containing bioplastic (CNC-BP) showed the highest elongation at break (100%) and the lowest thickness-normalized optical attenuation (0.38 mm^−1^), whereas the α-cellulose-containing bioplastic (α-C-BP) exhibited the highest maximum degradation-rate temperature (approximately 315 °C). Increasing the BC content from 0.25 to 1.0 g increased tensile strength from 3.17 ± 0.13 to 7.78 ± 0.31 MPa and Young’s modulus from 0.260 ± 0.002 to 0.996 ± 0.009 MPa. This increase was accompanied by a reduction in elongation at break from 41 ± 1.6% to 16 ± 0.6%. The BC-BP films retained their visible integrity after exposure to selected organic solvents but underwent substantial changes under strongly acidic and alkaline conditions. Following thermally induced repair, the BC-BP film recovered approximately 55% of its tensile strength and 45% of its Young’s modulus while retaining an elongation at break close to that of the original film. These results demonstrate that cellulose type and BC content can be used to adjust the measured thermal, optical, mechanical, and repair properties of starch–cellulose–Laponite films. Further structural, barrier, migration, and food-contact safety evaluations are required to establish their suitability for packaging applications.

## 1. Introduction

The widespread use of petroleum-derived plastics has created major environmental concerns because of their persistence, accumulation in landfills, and contribution to microplastic pollution [1,2]. Consequently, increasing attention has been directed toward biodegradable materials prepared from renewable polymers [3,4]. Starch is a promising matrix for bioplastic production because of its abundance, low cost, and biodegradability [5,6]. However, native starch-based films generally exhibit high water sensitivity, insufficient mechanical strength, and limited thermal stability, restricting their practical use in packaging applications [7,8,9].

Cellulose-based fillers have been investigated as reinforcements for improving the performance of starch-based materials [10,11,12]. Their reinforcing effects depend not only on cellulose content but also on particle dimensions, morphology, crystallinity, surface chemistry, and compatibility with the polymer matrix. Bacterial cellulose (BC) possesses a three-dimensional nanofibrillar architecture, high crystallinity, and numerous surface hydroxyl groups that can promote hydrogen bonding and mechanical stress transfer within starch matrices [13,14]. In comparison, α-cellulose consists of larger and more crystalline particles, whereas carboxylated cellulose nanofibers contain nanoscale fibrils and surface carboxyl groups that may enhance dispersion and interfacial interactions. Therefore, these cellulose forms are expected to influence film organization and properties through different reinforcement mechanisms.

Laponite is a synthetic layered silicate with nanoscale platelet morphology and a high specific surface area [15,16]. Its surface groups can interact with hydroxyl-containing polymers and modify polymer-chain mobility, thermal behavior, and barrier properties [16]. Previous studies have separately demonstrated the reinforcing effects of cellulose materials or Laponite in starch-based films [14,15]. However, direct comparison of BC, α-cellulose, and carboxylated cellulose nanofibers at comparable loading within the same starch–Laponite–glycerol matrix remains limited. Consequently, it is unclear how differences in cellulose morphology and surface functionality affect intermolecular interactions and the resulting mechanical, thermal, and optical properties under otherwise similar formulation conditions. This represents the principal knowledge gap addressed in the present study.

We hypothesized that differences in the morphology and surface functionality of cellulose reinforcements would produce distinct property profiles in starch–Laponite composite films. BC was expected to provide effective mechanical reinforcement through its interconnected nanofibrillar structure, whereas carboxylated cellulose nanofibers were expected to promote improved dispersion through their nanoscale dimensions and surface carboxyl groups. The comparatively larger and more crystalline α-cellulose particles were expected to affect thermal stability and optical behavior differently from the nanoscale fillers. Accordingly, the purpose of this study was to compare the effects of BC, α-cellulose, and carboxylated cellulose nanofibers on the intermolecular interactions, thermal stability, optical transparency, and mechanical properties of corn-starch–Laponite films and to evaluate the influence of BC content on mechanical performance, swelling, solvent resistance, and thermally induced repair.

## 2. Materials and Methods

### 2.1. Materials

Bacterial cellulose (BC) was purchased from Alfa Chemistry (Ronkonkoma, NY, USA) and supplied as an aqueous gel with a nominal cellulose content of approximately 2 wt.%. According to the manufacturer’s product specification, BC is produced by microbial fermentation of sugars and consists of high-aspect-ratio cellulose fibers with diameters of 50–100 nm and lengths exceeding 20 μm. The material has a cellulose I crystalline structure and hydroxyl-functionalized surfaces. The manufacturer also reports a water-absorption capacity of approximately 200 times its dry mass. The solid content of the supplied BC gel was experimentally determined by gravimetric oven drying as 2.94 ± 0.34 wt.% (*n* = 3). Therefore, the BC amounts used in all formulations were calculated on a dry-cellulose basis.

Corn starch was purchased from LLP “Zharkent Starch Factory” (Zharkent, Zhetysu Region, Kazakhstan). Glycerol (≥99.5%, CAS 56-81-5), Laponite (CAS 53320-86-8), α-cellulose (particle size 50–400 μm, CAS 9004-34-6), and carboxylated cellulose nanofibers (fiber diameter 4–10 nm, CAS 9004-34-6) were purchased from Sigma-Aldrich (Gillingham, UK). Numerical purity values for the corn starch, Laponite, α-cellulose, and carboxylated cellulose nanofibers were not specified in the available supplier documentation. All materials were used as received without further purification.

### 2.2. Preparation of Bioplastics

Bioplastics were prepared using corn starch, cellulose components (BC/ CNC, /α-C) BC, glycerol, and Laponite as the main components. Corn starch (5 g) was dispersed in distilled water under continuous stirring. Predetermined amounts of wet BC gel were incorporated based on its experimentally determined solid content of 2.94 wt.%. The required mass of wet BC gel was calculated on a dry-cellulose basis using the following relationship (Equation (1)):

(1) $$m_{wet}BC=\frac{m_{dry}BC}{0.0294}$$

Accordingly, 8.5, 17.0, and 34.0 g of wet BC gel were used to provide 0.25, 0.50, and 1.00 g of dry BC in the Low, Mid, and High formulations, respectively. Subsequently, 2.0 mL of glycerol (99.5%) was added as a plasticizer.

Separately, a 1% *w*/*v* Laponite suspension was prepared by dispersing Laponite powder in distilled water using a high-speed homogenizer (AD500S-H, Hangzhou BOYN Instrument Co., Ltd., Hangzhou, China) at 20,000 rpm for 20 min). A fixed volume of 5.5 mL of the Laponite suspension was added to each formulation. The amount of distilled water in each batch was adjusted so that the total water, including the water present in the wet BC gel and the Laponite suspension, was approximately 100 mL (Table 1). Glycerol was not included in the calculation of the total water content. The mixtures were heated on a hotplate at 80–90 °C for 30 min under continuous stirring until starch gelatinization occurred. The hot viscous mass was cast into a 9 × 9 cm mold and spread evenly. The films were dried in an oven at 40 °C until constant mass. The dried films were carefully removed from the molds and conditioned at 23 ± 2 °C and 50 ± 5% relative humidity for 48 h before further characterization.

### 2.3. Determination of Water and Solid Content of BC

The moisture content of the commercial BC sample was determined using the gravimetric oven-drying method. Approximately 1 g of wet BC gel was weighed in pre-dried glass dishes and dried in a laboratory oven at 105 °C for approximately 24 h until constant mass was reached. After cooling in a desiccator, the samples were reweighed. The masses of the wet and dry BC were determined after subtracting the mass of the empty glass dish. The solid content was calculated using Equation (2):

(2) $$Solid\;content\;(wt.\%)=\frac{m_{dry}}{m_{wet}}\times 100\%$$

where $m_{wet}$ is the initial mass of the wet BC gel and $m_{dry}$ is the mass remaining after oven drying. The water content was calculated as 100 − solid content. Each measurement was performed in triplicate, and the results are expressed as the mean ± standard deviation. The solid content of the commercial BC gel was 2.94 ± 0.34 wt.% (*n* = 3), corresponding to a water content of 97.06 ± 0.34 wt.%.

### 2.4. Optical Transparency Measurement

The optical transparency of the bioplastic films was determined using a UV–Vis spectrophotometer (λ = 600 nm). Film specimens were cut into 1 × 3 cm strips and directly placed in the sample holder without cuvettes. The transmittance (T_600_) values were recorded, and film thickness (d) was measured with a digital micrometer at five random points. The wavelength of 600 nm was selected as a representative point in the visible region, where absorption by the principal film components is expected to be relatively low. Consequently, differences in transmittance at this wavelength primarily reflect visible-light scattering associated with filler dispersion, aggregation, and internal film structure. The measurement was intended to compare the relative optical transparency of the prepared films rather than to characterize their wavelength-dependent UV-blocking properties. The transparency parameter (T) was calculated according to Equation (3):

(3) $$Transparency=-\frac{{log}_{10}(T_{600})}{d}$$

where *T*_600_ is the fractional transmittance at 600 nm, expressed as a value between 0 and 1, and *d* is the film thickness (mm). When transmittance was recorded as a percentage, it was divided by 100 before calculation. Higher values of the calculated parameter indicate lower optical transparency.

### 2.5. Swelling Behavior of Bioplastics

The swelling behavior of the bioplastic films was examined in distilled water at room temperature. The swelling degree was calculated using Equation (4):

(4) $$Swelling=\frac{m_{t}-m_{0}}{m_{0}}\times 100\%$$

where $m_{0}$ and $m_{t}$ represent the initial and swollen masses, respectively. All measurements were performed in triplicate (*n* = 3).

### 2.6. Solvent Resistance Test

The solvent resistance of the BC-BP bioplastics was evaluated by immersing rectangular film specimens (10 × 10 mm) in tetrahydrofuran (THF), toluene, chloroform, 1 M HCl, and 1 M NaOH at room temperature for 24 h. After immersion, the samples were removed, gently blotted with filter paper, and dried at 50 °C to constant weight. The treated films were subsequently examined for changes in morphology, color, and structural integrity and compared with the pristine samples to assess their chemical stability.

### 2.7. Tests Tensile Testing and Mechanical Characterization

The mechanical properties of the bioplastic films were evaluated using a TA.XT Plus texture analyzer (Stable Micro Systems, Surrey, UK) equipped with A/TG tensile grips, following a procedure adapted from ASTM D882 [17]. Rectangular film specimens measuring 20 × 20 mm were mounted between the tensile grips and tested at room temperature. The initial thickness of each specimen was measured at five randomly selected positions using a digital micrometer, and the mean thickness was used to calculate the initial cross-sectional area. The specimens were stretched at a crosshead speed of 2 mm s^−1^ until failure. Three specimens were tested for each formulation (*n* = 3).

Tensile strength was calculated as the maximum force divided by the initial cross-sectional area of the specimen. Elongation at break was calculated as the percentage increase in specimen length at failure relative to the initial gauge length. Young’s modulus was determined from the slope of the initial linear region of the stress–strain curve. The results are presented as the mean ± standard deviation.

### 2.8. Self-Healing and Reprocessing Capability Tests

The self-healing performance of the BC-BP bioplastics was evaluated by cutting the films into two pieces using a sharp blade. The cut surfaces were carefully brought into contact, aligned, and placed between Teflon sheets, followed by hot pressing at 60 °C for 60 s under moderate pressure using a hot press (Hangzhou BOYN Instrument Co., Ltd., Hangzhou, China). The repaired films were tested using the same tensile-testing procedure described in Section 2.7. Their tensile strength, elongation at break, and Young’s modulus were compared with those of the corresponding pristine films to evaluate the recovery of mechanical properties.

### 2.9. Fourier Transform Infrared (FTIR) Spectroscopy

FTIR spectra of the samples were recorded using a Cary 660 FTIR spectrometer (Agilent Technologies, Santa Clara, CA, USA). The spectra were collected from an average of 16 scans over the range of 4000–700 cm^−1^ in absorbance mode with a spectral resolution of 4 cm^−1^. The obtained spectra were processed and analyzed using OriginPro 2024b (OriginLab Corporation, Northampton, MA, USA).

### 2.10. Thermogravimetric Analysis (TGA)

Thermogravimetric analysis (TGA) was performed using a LabSys Evo thermogravimetric analyzer (SETARAM Instrumentation, Caluire-et-Cuire, France). Samples (8–10 mg) were heated from 30 to 600 °C at a heating rate of 10 °C min^−1^ under a nitrogen atmosphere. Thermograms were plotted and analyzed using OriginPro 2024b (OriginLab Corporation, Northampton, MA, USA).

### 2.11. Dynamic Light Scattering (DLS)

Dynamic light scattering measurements were carried out using a Zetasizer Nano ZS90 (Malvern Panalytical, Malvern, UK). The zeta potential of laponite was determined, and the corresponding results are provided in the Supporting Information.

### 2.12. SEM Analysis

Samples were mounted on aluminum stubs (Ø12 or 25 mm) using conductive carbon adhesive tape, and excess particles were removed with a gentle stream of dry air. No conductive coating was applied. Imaging was performed on a JEOL JSM-7000 scanning electron microscope (JEOL Ltd., Tokyo, Japan) in low-vacuum mode at a chamber pressure of 10–30 Pa and a working distance of ~10 mm. Topographic contrast was obtained with a low-vacuum secondary electron (LV-SE) detector at 5–10 kV, while compositional contrast was recorded using a backscattered electron (BSE) detector at 10–15 kV. Images were acquired at magnifications ranging from 100× to 20,000×. Representative SEM images are provided in the Supporting Information.

## 3. Results

**Figure 1:**
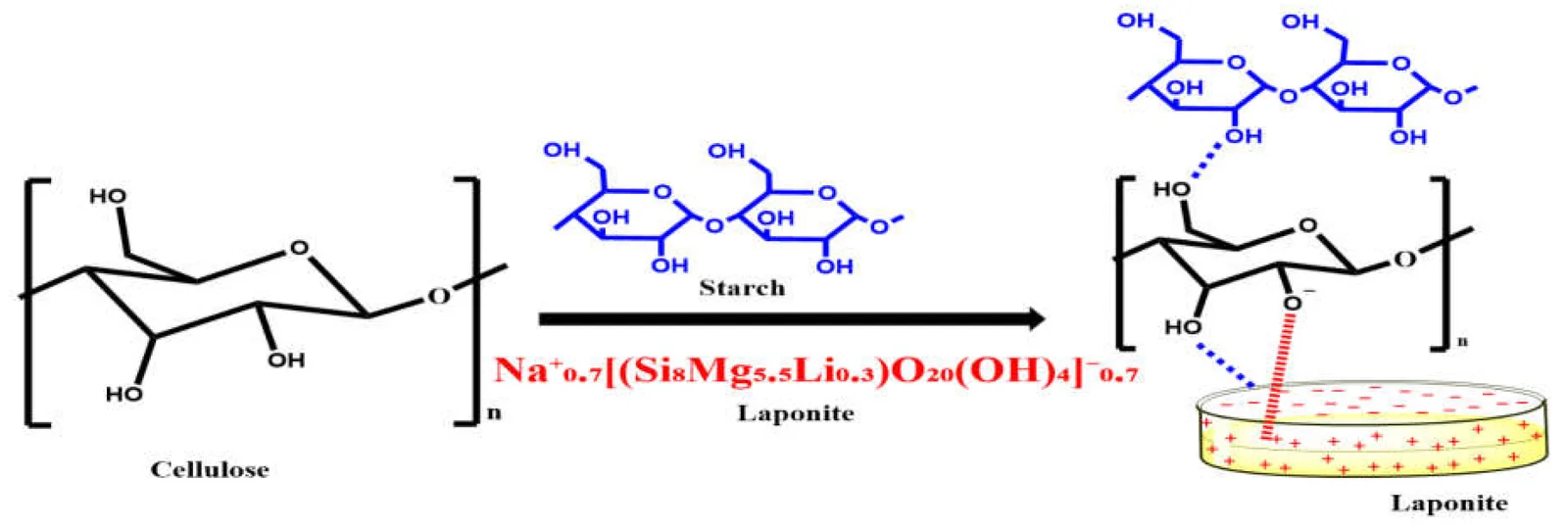
Schematic representation of the potential physical associations among starch, cellulose reinforcements, glycerol, and Laponite in the composite films. The plus sign indicates the combination of the components, while the positive and negative signs in the Laponite formula denote ionic charges. Black, blue, and yellow distinguish cellulose, starch, and Laponite, respectively, while the dotted lines represent proposed intermolecular interactions. The illustrated hydrogen bonding, electrostatic association, and chain entanglement represent proposed mechanisms and were not directly validated in the present study.

**Figure 2:**
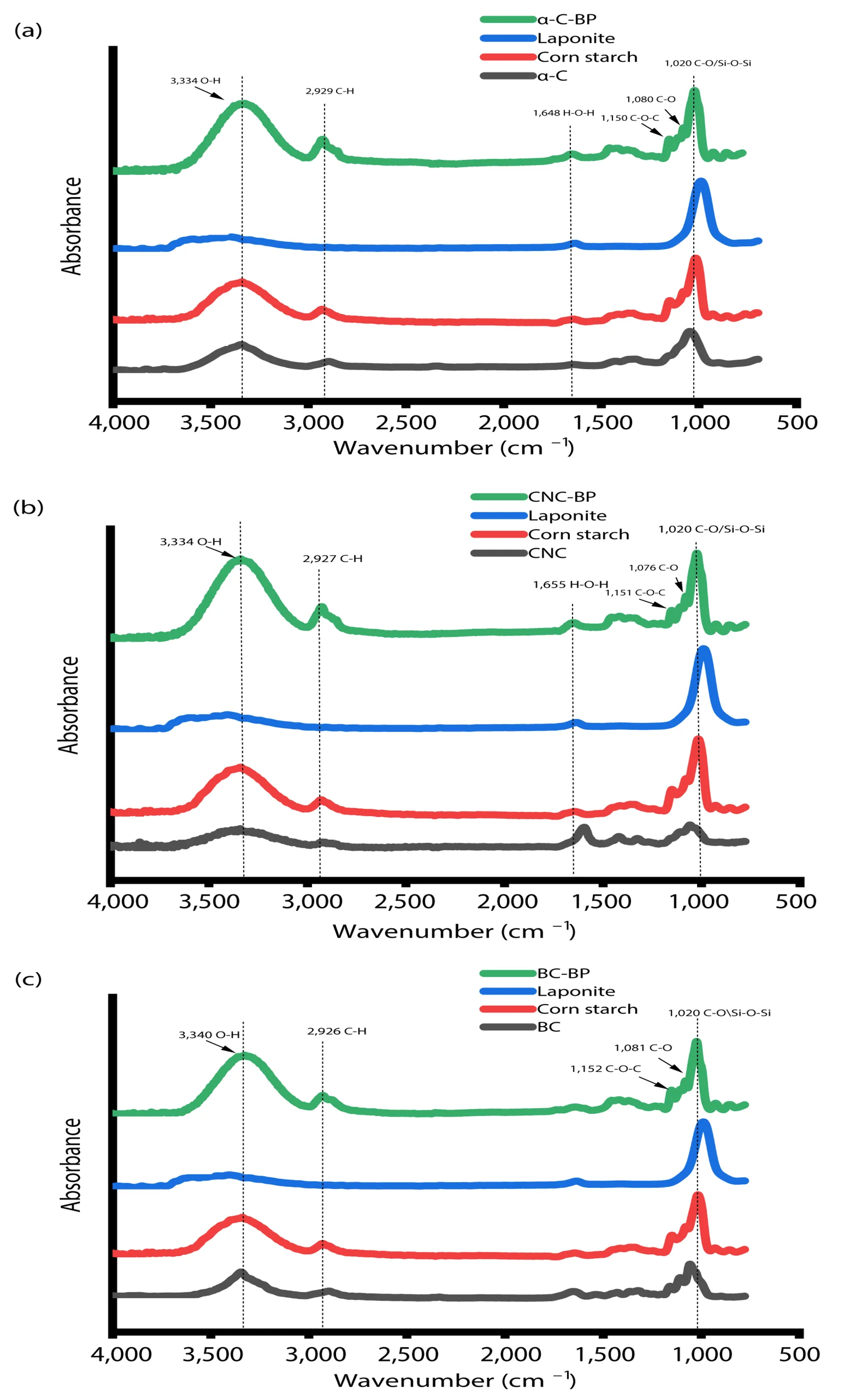
FTIR spectra of (**a**) α-C-BP, (**b**) CNC-BP, and (**c**) BC-BP composites and their corresponding components, highlighting variations in O–H, C=O/COO^−^, and Si–O bands associated with hydrogen bonding, electrostatic interactions, and laponite dispersion within the polymer matrix.

The composite structure may involve different physical association pathways between starch, Laponite, and the respective cellulose fillers, as schematically proposed in Figure 1. The FTIR spectra of corn starch-based bioplastics reinforced with α-cellulose (α-C-BP), carboxylated cellulose nanofibers (CNC-BP), and bacterial cellulose (BC-BP) in the presence of Laponite and glycerol are presented in Figure 2a–c. All composite spectra exhibit a broad absorption band within the narrow range of 3334–3340 cm^−1^, assigned to O–H stretching vibrations [13,16,18,19]. Considering the spectral resolution of 4 cm^−1^ and the absence of replicate FTIR measurements and an unreinforced reference matrix, the small differences in peak position were not treated as quantitative evidence of changes in hydrogen-bond strength or hydrogen-bond-network formation. The presence of this band is consistent with hydroxyl-containing components capable of participating in hydrogen bonding; however, it does not independently establish the extent or specific configuration of such interactions [13,16,18]. The bands at 2926–2929 cm^−1^ are attributed to C–H stretching vibrations of the polysaccharide backbones [19].

The absorption bands at 1648–1655 cm^−1^ are mainly associated with H–O–H bending vibrations of absorbed water, reflecting the hydrophilic character of the composites [19,20,21,22]. In CNC-BP (Figure 2b), this region may additionally contain a contribution from asymmetric stretching vibrations of carboxylate groups, although this assignment should be considered together with the corresponding symmetric COO^−^ vibration in the 1459–1413 cm^−1^ region [16]. The bands at 1459–1413 cm^−1^ are primarily attributed to CH_2_ bending vibrations and, in CNC-containing samples, may also include symmetric COO^−^ stretching [16]. The bands at 1367–1339 cm^−1^ arise from C–H deformation and O–H bending vibrations [16,23].

Strong absorption bands in the 1152–1076 cm^−1^ region are assigned to C–O–C and C–O stretching vibrations of the polysaccharide structures [16,23]. The intense band at approximately 1020 cm^−1^ contains overlapping contributions from C–O vibrations of starch and cellulose and Si–O–Si stretching vibrations of Laponite, consistent with the presence of the nanoclay in the composite formulation [13,16,18]. The band near 926 cm^−1^ is associated with the α-1,4-glycosidic linkages of starch, whereas the bands at 853–857 cm^−1^ are attributed to C–O/C–O–C and skeletal vibrations of the polysaccharide structure rather than exclusively to a characteristic Si–O vibration of Laponite [13,16,23]. Overall, the preservation of the characteristic component bands and the observed variations in band position, shape, and intensity are consistent with physical association among the composite components [16,23]. Nevertheless, FTIR alone cannot conclusively establish hydrogen-bonding configurations, electrostatic interactions, chain entanglement, or the resulting network architecture.

**Figure 3:**
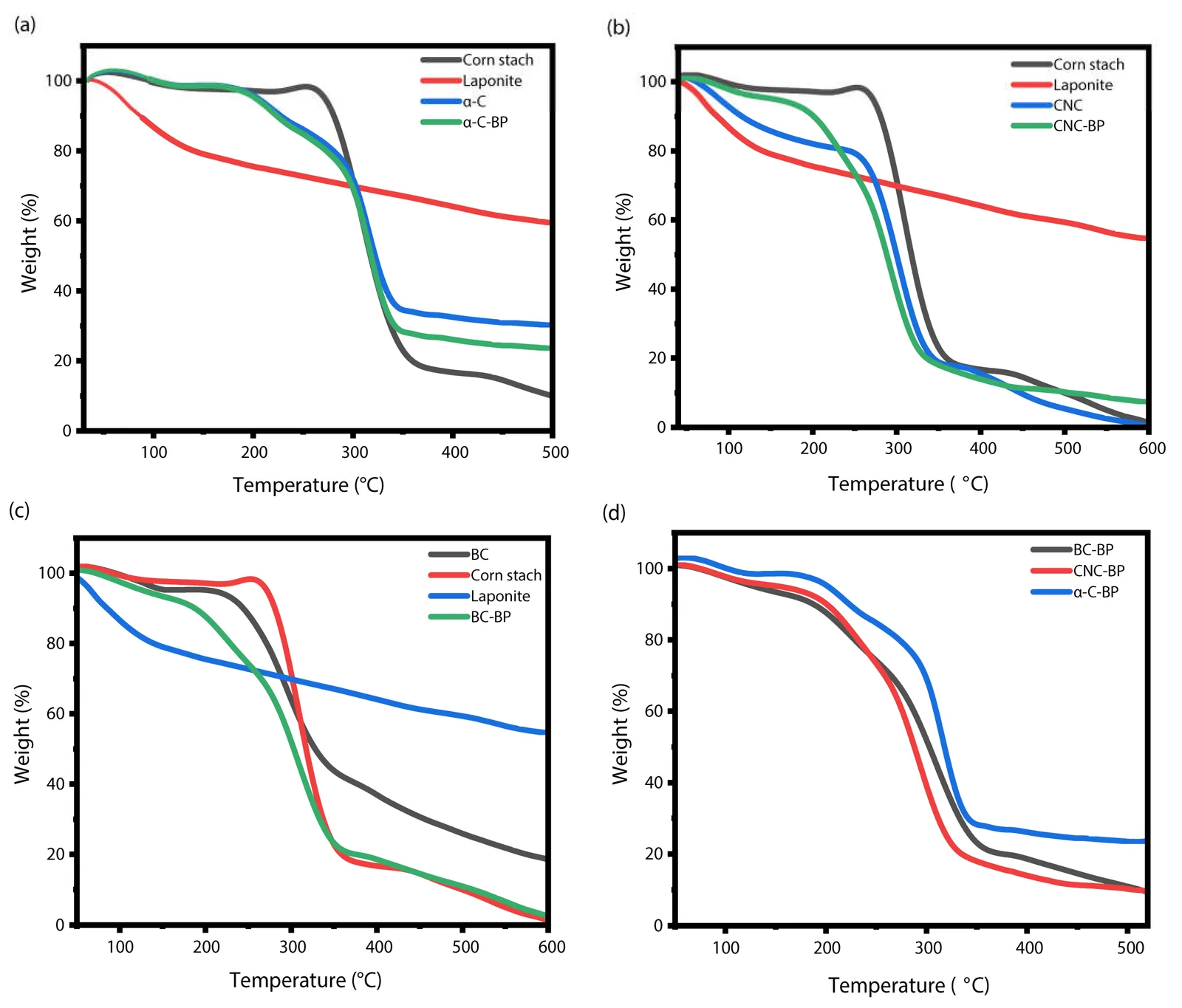
Thermogravimetric analysis (TGA) of starch-based bioplastic systems. (**a**) TGA curves of α-C-BP and corresponding components (corn starch, laponite, and α-cellulose); (**b**) TGA curves of CNC-BP and corresponding components; (**c**) TGA curves of BC-BP and corresponding components; (**d**) TGA curves of composite films (α-C-BP, CNC-BP, and BC-BP).

**Figure 4:**
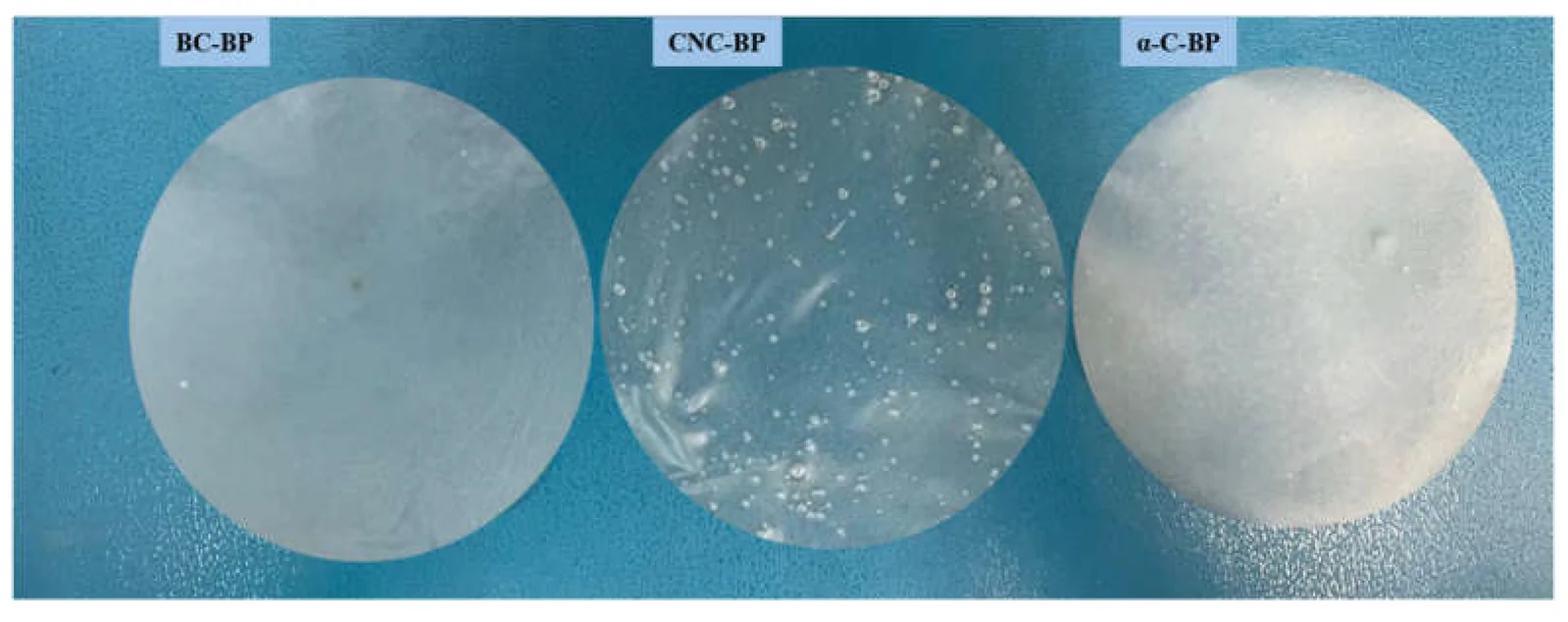
Photographs of starch-based bioplastic films reinforced with bacterial cellulose (BC-BP), carboxylated cellulose nanofibers (CNC-BP), and α-cellulose (α-C-BP).

The thermogravimetric behavior of starch-based bioplastics reinforced with α-cellulose (α-C-BP), bacterial cellulose (BC-BP), and carboxylated cellulose nanofibers (CNC-BP) in the presence of laponite and glycerol reveals a characteristic multi-step degradation profile typical of polysaccharide-based composites (Figure 3a–c). An initial weight loss below 120 °C corresponds to the removal of physically adsorbed and bound water, which is consistent with the water affinity of the polymer–clay matrices, while the main degradation stage occurs within 250–350 °C due to depolymerization, dehydration, and cleavage of glycosidic bonds in starch and cellulose, accompanied by glycerol volatilization. The DTG curves exhibit single dominant peaks corresponding to the maximum degradation rate (T_max_), with values of approximately 295 °C (BC-BP), 305 °C (CNC-BP), and 315 °C (α-C-BP) (Table 2). The higher T_max_ of α-C-BP indicates greater resistance to thermal degradation under the tested conditions, whereas CNC-BP and BC-BP exhibit intermediate and lower T_max_ values, respectively. However, TGA alone cannot determine whether these differences originate from cellulose crystallinity, nanoscale filler dispersion, hydrogen bonding, or other structural interactions. BC-BP exhibits a comparatively gradual mass-loss profile; however, this observation cannot independently demonstrate the formation of a stable or well-integrated polymer–nanoclay network. At temperatures above 400 °C, all samples exhibit slow degradation associated with carbonaceous residue decomposition. These trends are consistent with the macroscopic appearance of the films (Figure 4), where BC-BP exhibits a visually uniform morphology comparable to those of the other systems. Nevertheless, macroscopic appearance and TGA results cannot confirm nanoscale dispersion, hydrogen bonding, or fibrillar entanglement. Direct morphological characterization, such as TEM analysis, would be required to evaluate filler dimensions and dispersion within the starch matrix.

The optical properties of the starch-based bioplastics were evaluated to compare their thickness-normalized light-transmission behavior at 600 nm (Table 3). The calculated optical parameter reflects attenuation of transmitted light through a combination of absorption and scattering. Although scattering may be influenced by filler size, aggregation, refractive-index contrast, surface roughness, and film thickness, a single-wavelength optical measurement cannot independently determine filler dispersion, structural homogeneity, or interfacial compatibility [12,15,16,18]. Among the investigated systems, CNC-BP exhibited the highest transparency, with the lowest calculated parameter of 0.38 mm^−1^ and a transmittance of 75.5%. This result demonstrates lower optical attenuation at 600 nm relative to α-C-BP and BC-BP but does not establish homogeneous nanoscale distribution of the carboxylated cellulose nanofibers. In contrast, α-C-BP and BC-BP exhibited higher calculated optical parameters of 0.74 and 0.84 mm^−1^, respectively. These differences indicate greater thickness-normalized optical attenuation but cannot, without direct microscopic evidence, be attributed specifically to larger cellulose domains or less uniform dispersion. Previous studies have shown that the optical properties of starch–cellulose and polymer–Laponite films may depend on filler dimensions, aggregation, film morphology, and refractive-index differences among the components [13,18,19]. However, the relative contributions of these factors were not determined in the present study. No direct relationship between the optical measurements and the TGA results was inferred because the two methods evaluate different material properties. Accordingly, the optical data are interpreted only as a comparative measure of light transmission at 600 nm and not as evidence of structural homogeneity, interfacial compatibility, or nanoscale reinforcement.

**Figure 5:**
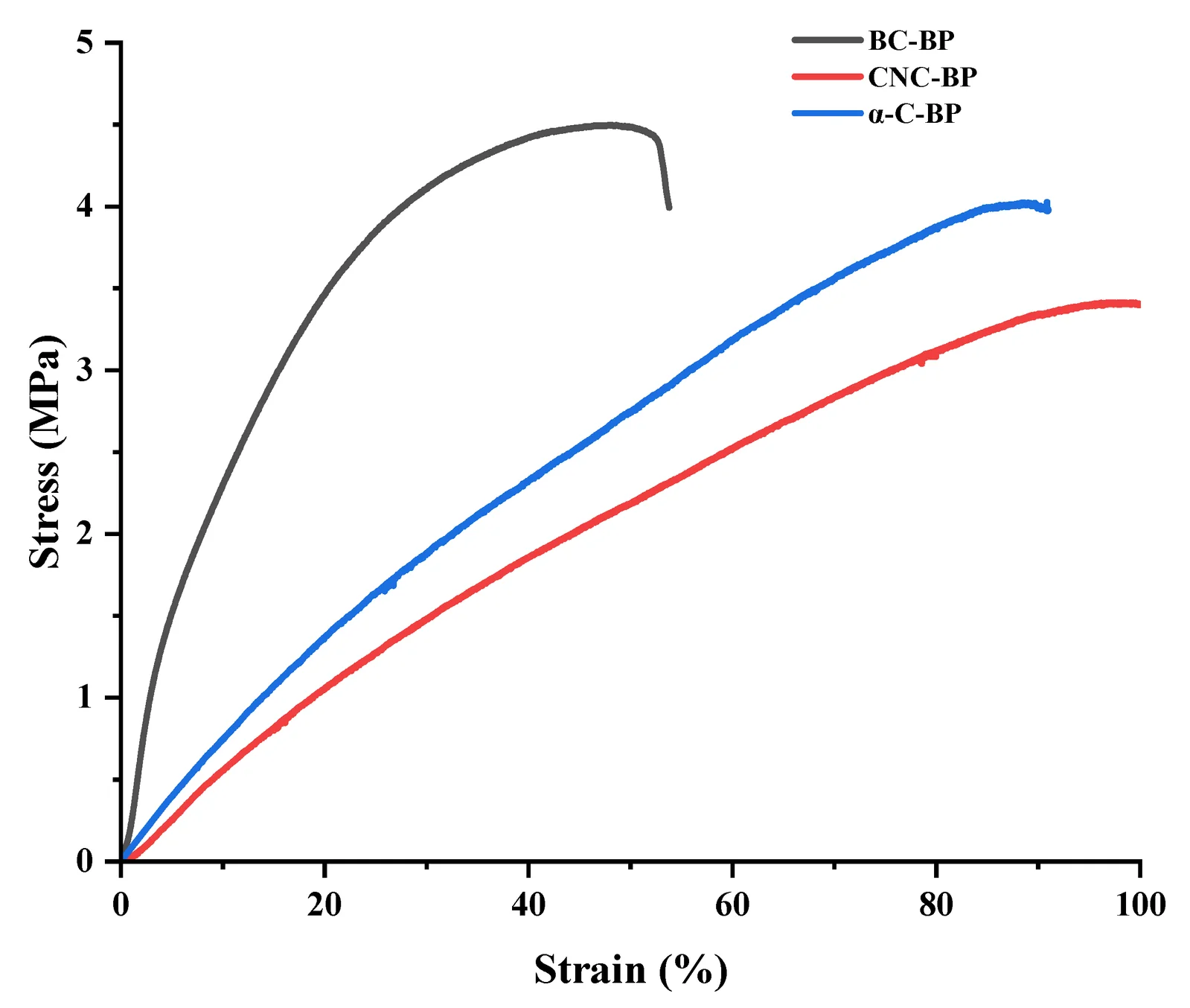
Stress–strain curves and mechanical properties of starch-based bioplastics reinforced with bacterial cellulose (BC-BP), carboxylated cellulose nanofibers.

The mechanical performance of the starch-based bioplastics strongly depends on the type of cellulose reinforcement, as evidenced by the stress–strain behavior (Figure 5) and summarized mechanical parameters (Table 4). Among the investigated systems, BC-BP exhibited the highest tensile strength (4.47 MPa) and Young’s modulus (0.229 MPa), indicating that the interconnected nanofibrillar network of bacterial cellulose effectively reinforces the starch matrix through extensive hydrogen bonding and physical entanglement. The α-C-BP film showed slightly lower tensile strength (4.00 MPa) while maintaining relatively high elongation at break (~89.7%), suggesting a more flexible network associated with the less ordered structure of α-cellulose fibers. In contrast, CNC-BP displayed the lowest tensile strength (3.40 MPa) but the highest elongation at break (~100%), reflecting enhanced ductility and more uniform stress distribution due to nanoscale dispersion of cellulose nanofibers. These results indicate that while nanoscale reinforcement improves flexibility, the continuous fibrillar morphology of bacterial cellulose provides more effective load transfer and overall mechanical reinforcement within the composite network.

**Figure 6:**
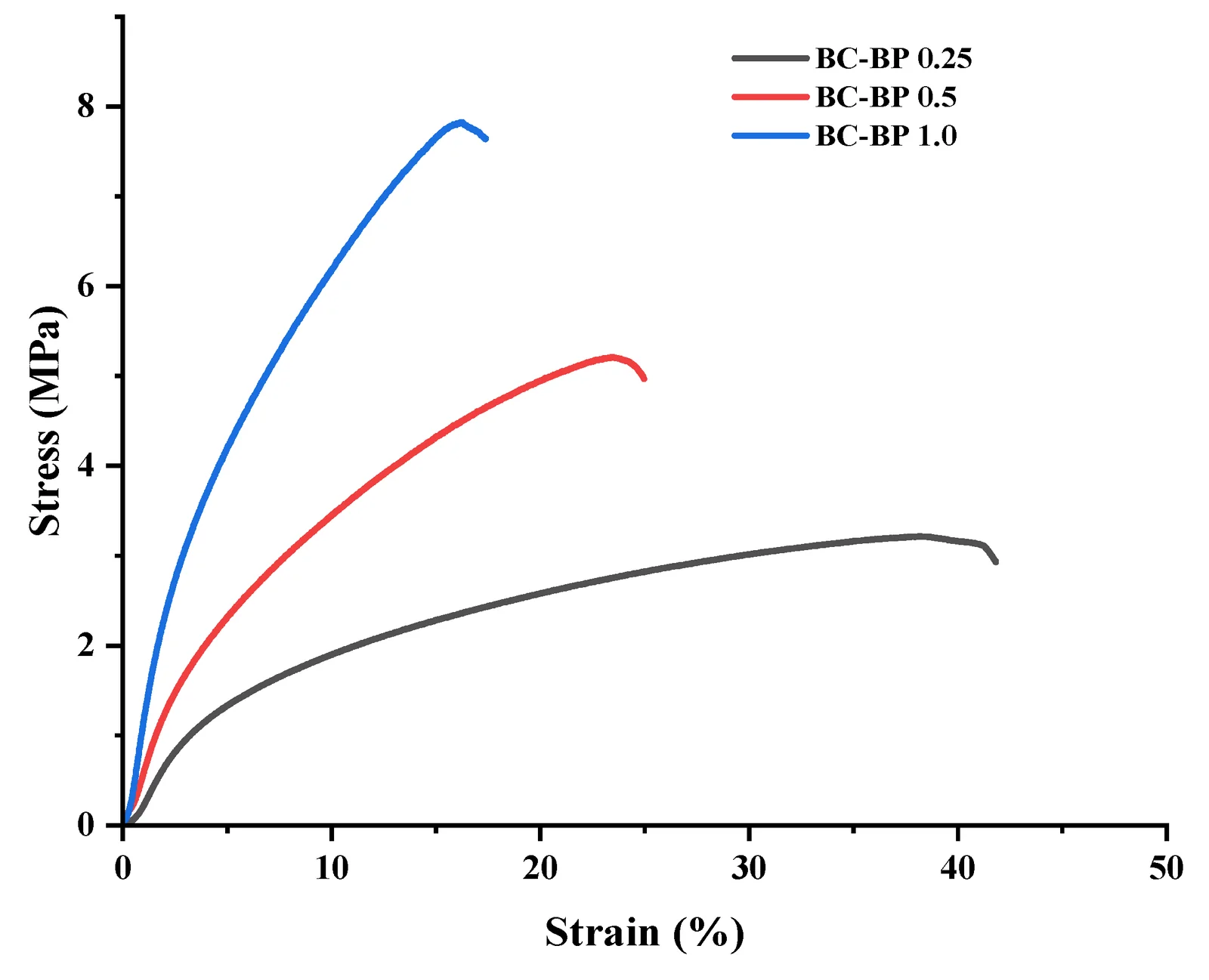
Stress–strain curves of BC-reinforced starch-based bioplastics containing different bacterial cellulose contents (0.25, 0.5, and 1.0).

**Figure 7:**
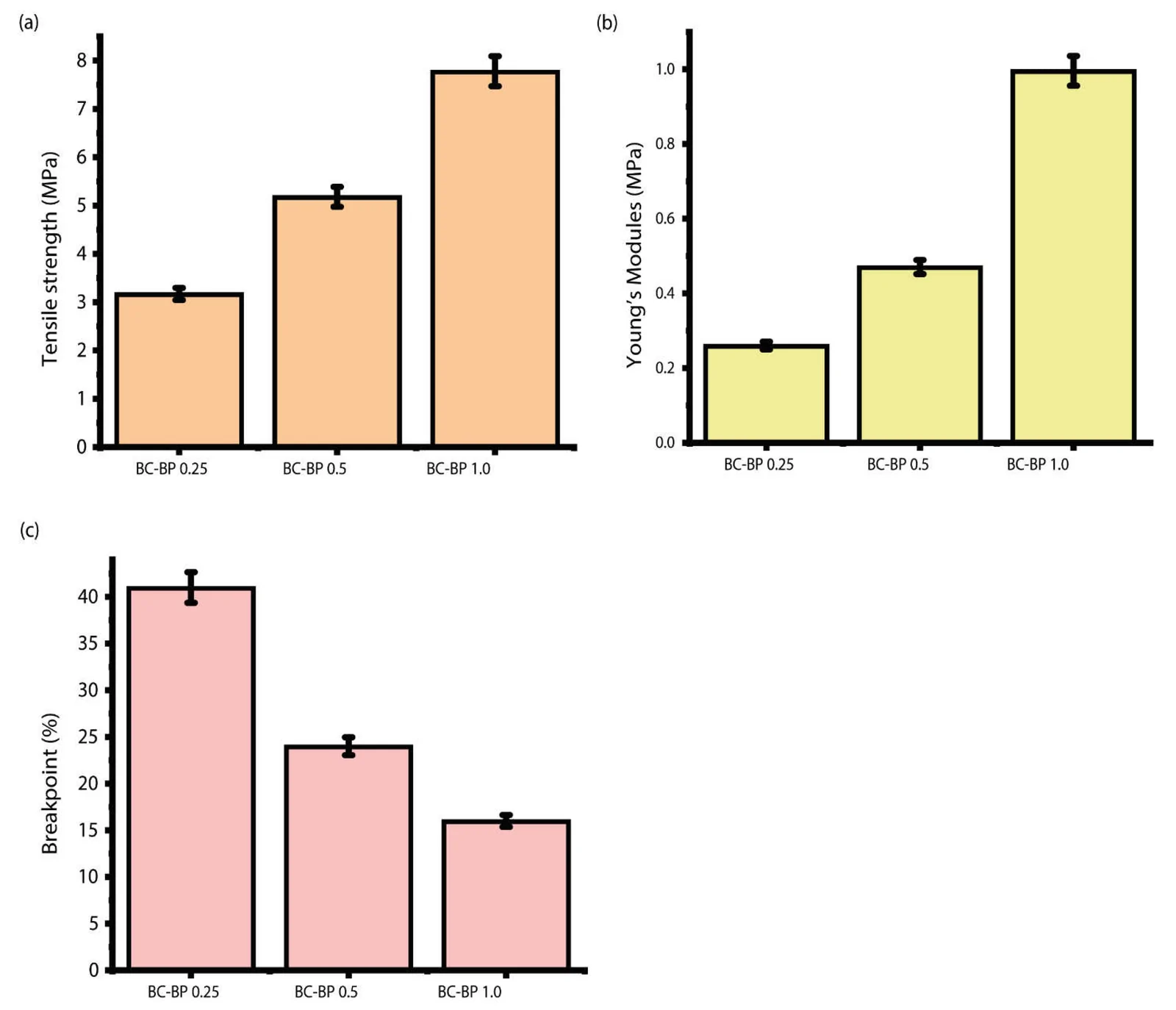
Mechanical properties of BC-reinforced bioplastics as a function of bacterial cellulose content: (**a**) tensile strength, (**b**) elongation at break, and (**c**) Young’s modulus.

The mechanical properties of BC-reinforced bioplastics strongly depend on bacterial cellulose content, as shown by the stress–strain behavior and summarized mechanical parameters (Figure 6 and Figure 7, Table 5). Increasing BC loading from 0.25 to 1.0 resulted in a pronounced improvement in tensile strength (from 3.17 to 7.78 MPa) and Young’s modulus (from 0.26 to 0.99 MPa), confirming the role of bacterial cellulose as an effective reinforcing nanofiller. This enhancement is attributed to the formation of a percolated nanofibrillar network that facilitates efficient stress transfer within the polymer matrix. In contrast, the elongation at break decreased from 41% to 16% with increasing BC content, indicating reduced flexibility and increased brittleness. This behavior is associated with restricted polymer chain mobility caused by strong hydrogen bonding and enhanced interfacial interactions between BC nanofibers and the starch matrix. At lower BC contents (0.25–0.5), the composites exhibit a balanced combination of strength and ductility, whereas higher loading (1.0) leads to increased stiffness at the expense of flexibility.

The swelling behavior of BC-BP bioplastic films demonstrates a time-dependent increase in water uptake, reaching an average swelling degree of ~182% after 24 h of immersion (Table 6). The most pronounced mass increase occurs within the first 6 h, followed by a gradual plateau, indicating the approach to swelling equilibrium. This behavior is attributed to the hydrophilic nature of the matrix components, including corn starch, bacterial cellulose, and glycerol, which contain abundant hydroxyl groups capable of forming hydrogen bonds with water molecules. The presence of laponite contributes to maintaining structural integrity by restricting excessive expansion of the polymer network during hydration. Despite variability among individual samples, the consistent increase in swelling degree and relatively stable standard deviation values indicate good reproducibility and confirm the formation of a stable hydrophilic polymer network.

**Figure 8:**
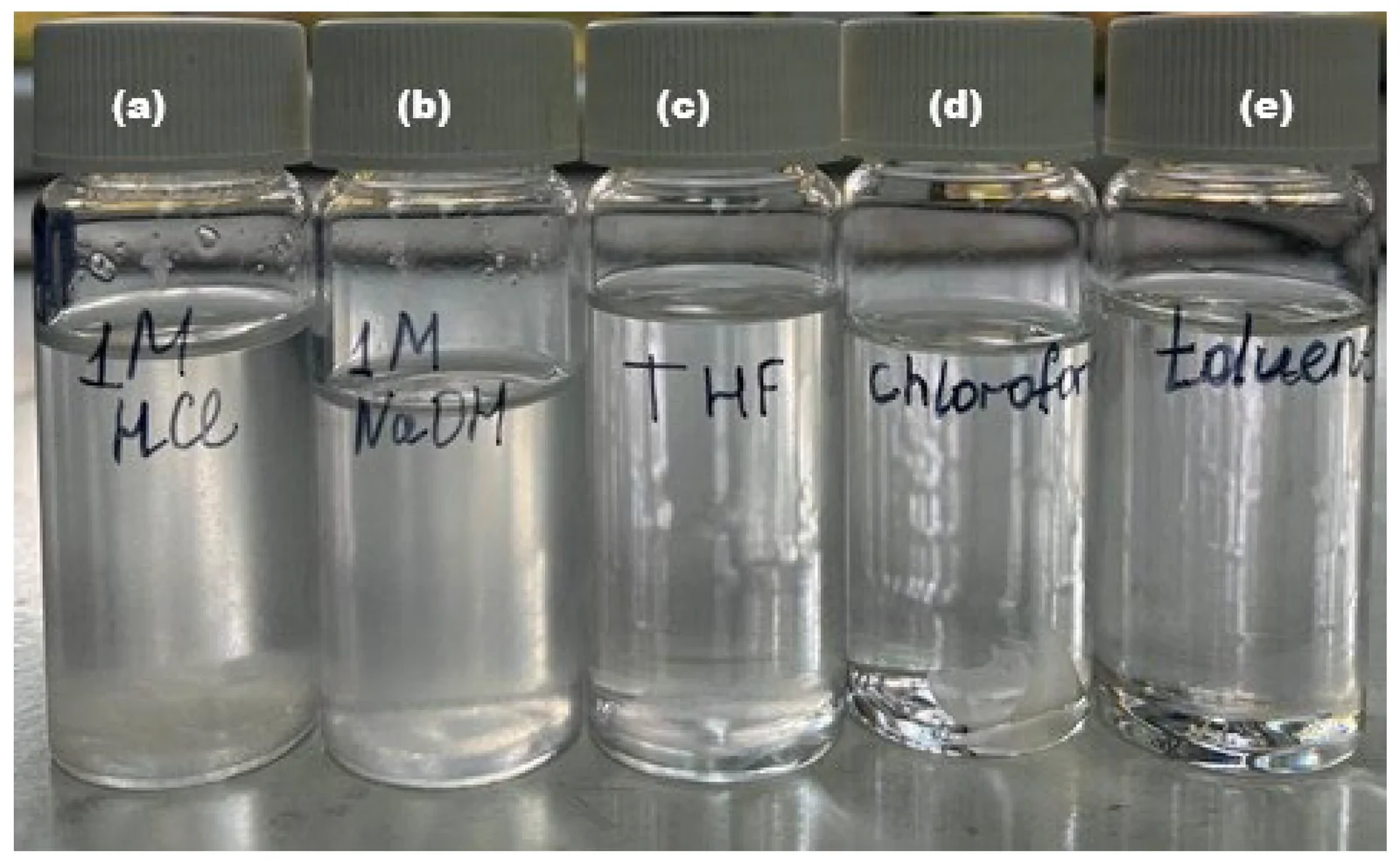
Visual appearance of BC-BP bioplastic films after 24 h immersion in different media at room temperature: (**a**) 1 M HCl, (**b**) 1 M NaOH, (**c**) THF, (**d**) chloroform, and (**e**) toluene. Films retain structural integrity in organic solvents but exhibit swelling and deformation under acidic and alkaline conditions.

The BC-BP bioplastic films exhibit pronounced chemical resistance toward organic solvents, retaining their shape, transparency, and structural integrity after 24 h of immersion in THF, chloroform, and toluene (Figure 8). This behavior indicates the formation of a stable polymer network governed by strong intermolecular hydrogen bonding and polymer–nanoclay interactions that limit solvent penetration. In contrast, exposure to acidic (1 M HCl) and alkaline (1 M NaOH) environments results in significant swelling, deformation, and partial dissolution of the films. Such degradation is attributed to hydrolytic cleavage of glycosidic linkages within the polysaccharide matrix under extreme pH conditions. The inability to reconstruct coherent films after complete dissolution further confirms irreversible structural disruption of the network. These results demonstrate that BC-BP bioplastics combine high resistance to organic solvents with pH-responsive degradability, highlighting their potential for environmentally triggered degradation and controlled end-of-life behavior.

**Figure 9:**
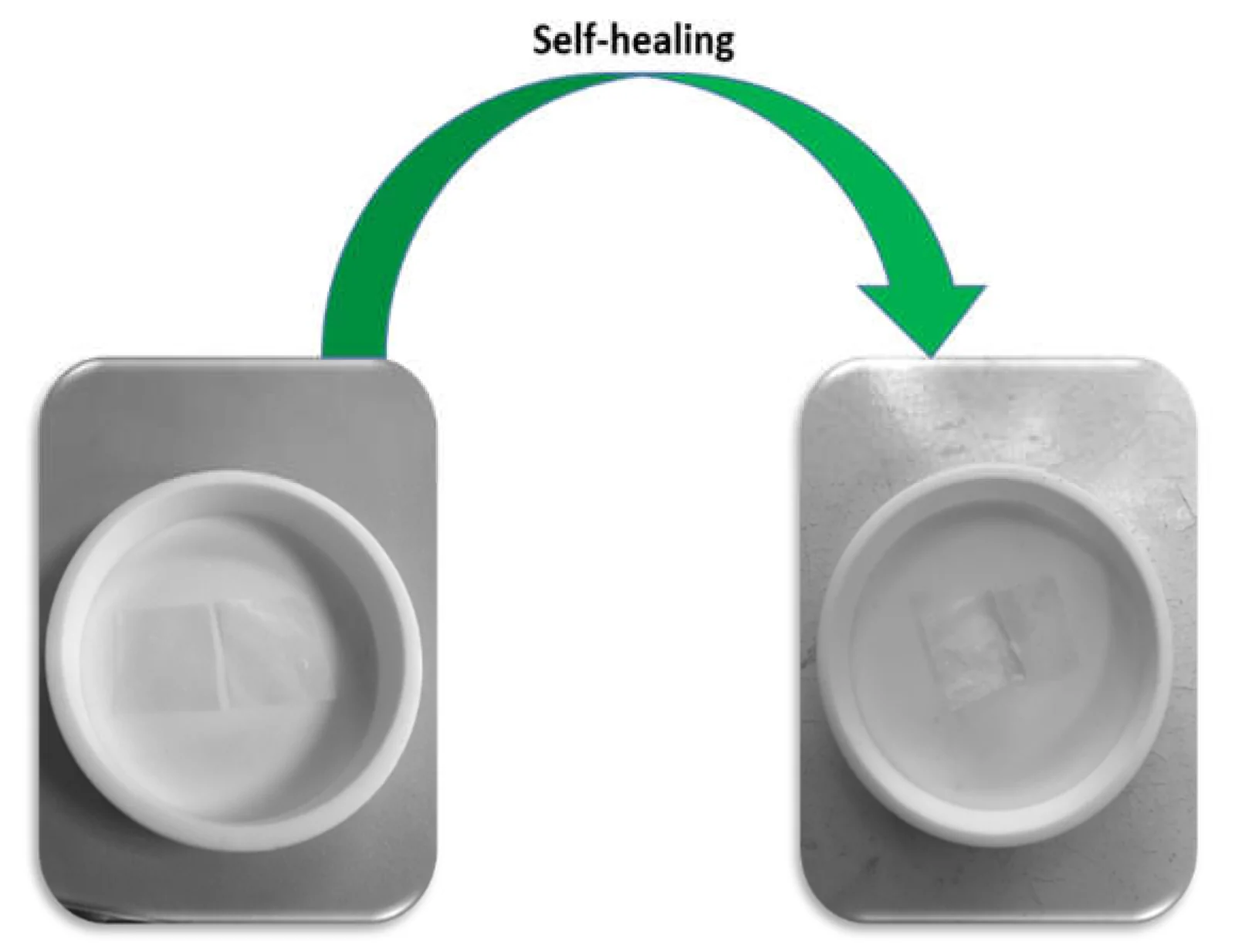
Thermally induced repair behavior of the BC-BP film, showing its appearance before repair (left) and after repair (right).

**Figure 10:**
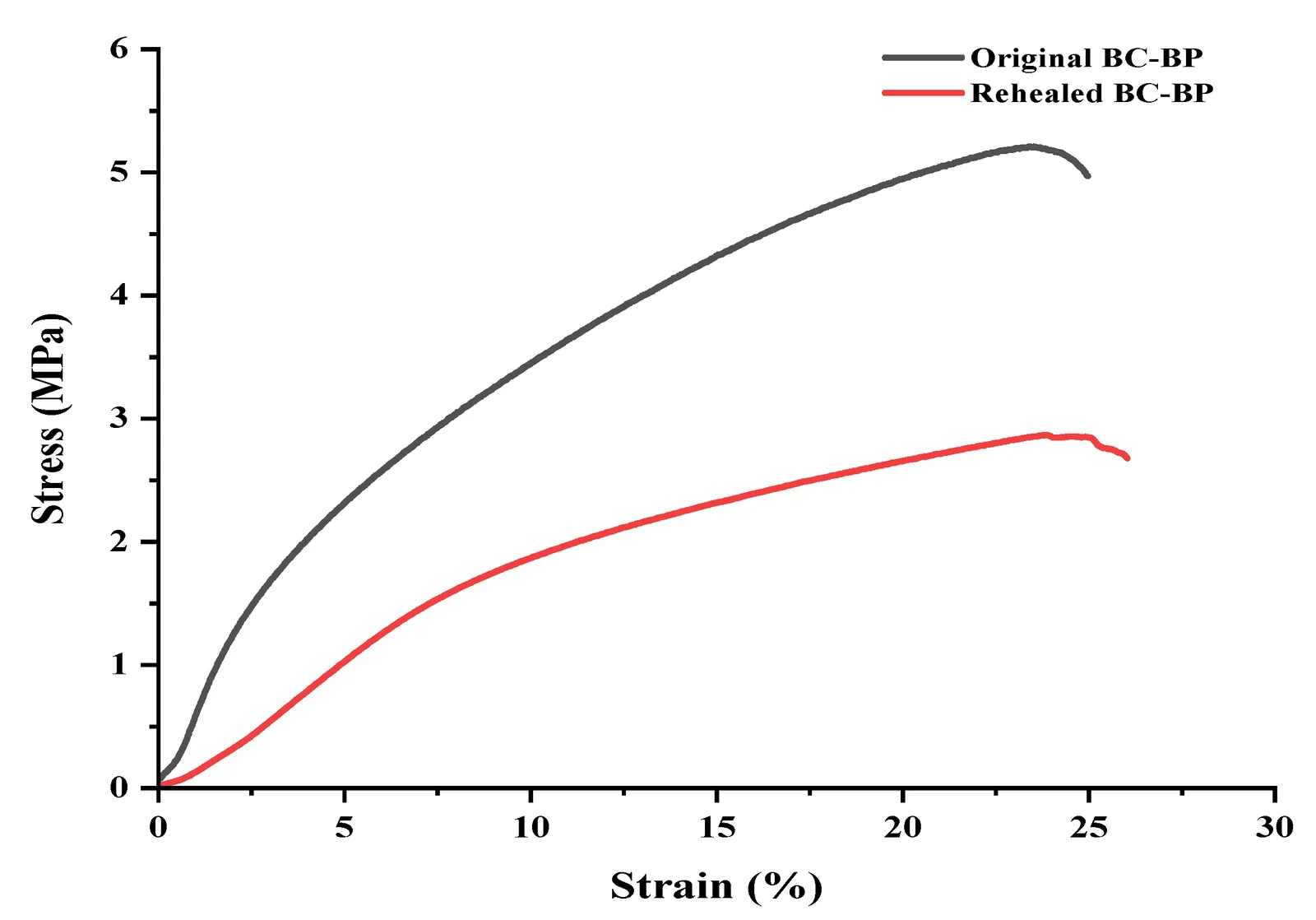
Stress–strain curves of original and rehealed BC-BP bioplastic films, showing partial recovery of mechanical properties after thermal healing.

The self-healing behavior of BC-BP bioplastics was evaluated by comparing the mechanical performance of pristine and rehealed films (Figure 9 and Figure 10, Table 7), where the original material exhibited a tensile strength of 5.18 MPa, elongation at break of 24%, and Young’s modulus of 0.471 MPa, while after thermal healing the tensile strength decreased to 2.85 MPa, elongation remained nearly unchanged (~23.7%), and Young’s modulus declined to 0.212 MPa, corresponding to ~55% recovery of strength and ~45% recovery of stiffness, indicating partial restoration of the polymer network integrity.

To provide a quantitative context for the mechanical performance of the developed films, their tensile properties were compared with those of previously reported starch-, cellulose-, and clay-based composite films (Table 8). Within the present BC-BP series, increasing the BC content from 0.25 to 1.0 g increased the tensile strength from 3.17 ± 0.13 to 7.78 ± 0.31 MPa and Young’s modulus from 0.260 ± 0.002 to 0.996 ± 0.009 MPa, while elongation at break decreased from 41 ± 1.6% to 16 ± 0.6%. The published formulations included in the comparison exhibited tensile strengths ranging from 4.885 to 71.32 MPa, elongation at break from 1.9 to 48.81%, and reported Young’s modulus values from 25.76 to 2530 MPa. Thus, the maximum tensile strength obtained in the present study was within the lower range of the published values. However, the formulations, film thicknesses, specimen geometries, conditioning procedures, crosshead speeds, and testing methods differed substantially among the studies.

## 4. Discussion

The results show that the type of cellulose incorporated into the starch–Laponite formulation was associated with different measured thermal, optical, and mechanical property profiles. Among the films containing different cellulose types, BC-BP exhibited the highest tensile strength and Young’s modulus, whereas CNC-BP showed the greatest elongation at break and the lowest thickness-normalized optical attenuation at 600 nm (Table 3 and Table 4). In the TGA analysis, α-C-BP exhibited the highest T_max_, followed by CNC-BP and BC-BP (Table 2). These observations demonstrate formulation-dependent differences among the three films but do not independently identify the structural mechanisms responsible for them. Because filler dispersion, filler dimensions within the films, cellulose crystallinity, interfacial adhesion, and polymer-chain mobility were not directly characterized, the measured differences cannot be conclusively attributed to nanoscale organization, interconnected polymer networks, or specific stress-transfer mechanisms. The following discussion therefore evaluates the experimentally observed FTIR, thermal, optical, and mechanical trends in the context of previous studies while treating the proposed structural explanations cautiously.

The FTIR spectra identified the characteristic functional groups of the starch, cellulose, glycerol, and Laponite components but did not independently establish the strength or configuration of their intermolecular interactions (Figure 2). The broad O–H band at approximately 3334–3340 cm^−1^ is consistent with the presence of hydroxyl-containing components capable of participating in hydrogen bonding, as commonly reported for biopolymer–clay systems [9,32,33]. However, the position and broadness of this band alone cannot confirm the formation or extent of an interconnected hydrogen-bonding network. The C–H stretching bands at approximately 2927–2929 cm^−1^ and the characteristic polysaccharide bands within the 1150–1020 cm^−1^ region indicate the retention of the principal starch and cellulose structures in the composite films [33,34,35]. In CNC-BP, the bands potentially associated with carboxylate groups are consistent with the presence of carboxylated cellulose [36,37,38]. Although these groups may provide sites for hydrogen bonding or electrostatic association, their specific interactions with Laponite cannot be demonstrated from the present FTIR spectra. Similarly, the overlapping C–O and Si–O–Si contributions near 1020 cm^−1^ are consistent with the presence of Laponite in the composite formulation but do not provide evidence of its nanoscale dispersion or direct participation in an interconnected network [34]. Previous studies have proposed hydrogen bonding and electrostatic association in comparable cellulose–starch–clay systems [39,40]; nevertheless, the present FTIR data do not permit the interaction strength to be ranked among CNC-BP, BC-BP, and α-C-BP. Additional analyses, such as solid-state NMR, rheological characterization, DMA, or SAXS, would be required to characterize these interactions and the resulting network architecture more directly.

The TGA results showed distinct degradation profiles for the three cellulose-containing films (Figure 3). The principal DTG peaks occurred at approximately 315 °C for α-C-BP, 305 °C for CNC-BP, and 295 °C for BC-BP (Table 2). Thus, α-C-BP exhibited the highest T_max_, whereas CNC-BP and BC-BP showed intermediate and lower values, respectively. Previous studies have shown that the thermal behavior of starch–cellulose composites can vary with filler type, cellulose crystallinity, dispersion, plasticizer content, and composite formulation [41,42]. However, the present TGA data cannot determine which of these factors caused the observed differences. The comparatively gradual mass-loss profile of BC-BP is an experimental observation, but it does not demonstrate the formation of a stable or interconnected polymer network. Although bacterial cellulose is commonly described as having a nanofibrillar architecture and relatively high crystallinity [43,44,45,46], these characteristics were not directly evaluated within the present films. Above 400 °C, all samples exhibited slow mass loss associated with further degradation of carbonaceous residues. The differences in residual mass may reflect variations in char formation and inorganic content, but they do not confirm the formation of carbon–silicate structures. Therefore, the TGA results describe differences in thermal degradation behavior but do not establish cellulose crystallinity, nanoscale reinforcement, filler dispersion, hydrogen bonding, interfacial adhesion, or polymer-chain mobility.

The optical measurements showed differences in thickness-normalized light attenu-ation at 600 nm among the three films (Table 3). CNC-BP exhibited the lowest calculated optical parameter of 0.38 mm^−1^. The corresponding values for α-C-BP and BC-BP were 0.74 and 0.84 mm^−1^, respectively. Optical attenuation in starch-based composite films may be affected by absorption, filler size, aggregation, refractive-index contrast, surface roughness, and film thickness [34,36,37,38]. However, these individual contributions were not determined in the present study. Therefore, the lower optical attenuation of CNC-BP cannot be interpreted as direct evidence of homogeneous nanoscale dispersion or stronger interfacial compatibility. Similarly, the higher values obtained for α-C-BP and BC-BP do not inde-pendently demonstrate filler aggregation or microstructural heterogeneity. Previous stud-ies have also reported composition-dependent optical behavior in starch–cellulose com-posite films [47,48,49,50]. Nevertheless, microscopy and wavelength-dependent optical meas-urements would be required to identify the structural origins of the observed differences.

The three cellulose-containing films exhibited different mechanical property profiles (Figure 5 and Table 4). BC-BP showed the highest tensile strength and Young’s modulus, with values of 4.47 and 0.229 MPa, respectively. Its elongation at break was 52.0%. Published studies indicate that the mechanical properties of starch–cellulose films can vary with filler type, filler content, plasticizer concentration, film thickness, and testing conditions [48,49,50]. The comparatively higher strength of BC-BP may therefore be associated with differences in its formulation and reinforcement form. However, the present mechanical data do not demonstrate an interconnected fibrillar network, efficient load transfer, or physical entanglement [51,52]. The α-C-BP film exhibited a tensile strength of 4.00 MPa and an elongation at break of 89.7%. These values cannot be attributed conclusively to α-cellulose crystallinity, dispersion, or interfacial adhesion because XRD and direct dispersion analyses were not performed [53,54]. CNC-BP showed the lowest tensile strength of 3.40 MPa and the highest elongation at break of 100%. This result indicates greater deformability under the applied testing conditions. It does not, however, establish homogeneous nanoscale dispersion or uniform stress distribution. Overall, the results demonstrate cellulose-dependent differences in mechanical behavior, while the mechanisms responsible for these differences require direct structural and morphological characterization.

Increasing the BC content produced clear concentration-dependent changes in the mechanical properties of the films (Figure 6 and Figure 7; Table 5). As the BC content increased from 0.25 to 1.0 g, tensile strength increased from 3.17 ± 0.13 to 7.78 ± 0.31 MPa and Young’s modulus increased from 0.260 ± 0.002 to 0.996 ± 0.009 MPa. Conversely, elongation at break decreased from 41 ± 1.6% to 16 ± 0.6%. These results demonstrate that increasing the BC content improved strength and stiffness but reduced film extensibility. Similar concentration-dependent behavior has been reported for other cellulose-reinforced biopolymer films [35,36,37,49,51,52]. In such systems, a higher cellulose content may increase the contribution of the reinforcing phase and reduce the deformability of the polymer matrix [53,54,55]. This interpretation is consistent with the observed strength–ductility trade-off. However, the specific contributions of BC distribution, interfacial adhesion, and fibrillar-network formation require direct morphological and structural characterization.

The maximum tensile strength obtained in the present study was 7.78 MPa, placing the developed films within the lower range of the published values summarized in Table 8. Comparable tensile strengths of approximately 4.7–5.8 MPa have been reported for several starch-based composite films [21,22,29,31]. However, other starch–cellulose formulations reached values between approximately 10 and 71 MPa [20,23,24,25,26,27,28,30]. The comparatively modest strength of the present films may partly reflect the plasticizing effect of glycerol. Glycerol facilitates film formation and flexibility but can also reduce cohesive strength by increasing polymer-chain mobility. In addition, the present formulation relied mainly on physical association among starch, BC, glycerol, and Laponite rather than deliberate covalent crosslinking. This formulation may therefore favor processability, extensibility, and thermally induced repair over maximum tensile strength. The hydrophilic character of the starch–cellulose matrix may also make its mechanical behavior sensitive to moisture and conditioning conditions. Nevertheless, direct numerical comparison remains limited because the published studies used different formulations, film thicknesses, specimen geometries, conditioning procedures, crosshead speeds, and testing standards. Further optimization of the glycerol content, BC loading, and processing conditions may improve tensile strength while maintaining adequate film flexibility.

The BC-BP films retained their visible shape and general appearance after 24 h of exposure to THF, chloroform, and toluene. These qualitative observations indicate limited macroscopic disruption under the applied test conditions. Similar behavior has been reported for polysaccharide–clay materials exposed to nonpolar organic solvents [56,57]. However, retention of visible integrity does not demonstrate a densely crosslinked network or restricted solvent diffusion. Quantitative measurements of solvent uptake, mass loss, and dimensional change would be required to establish solvent resistance. In contrast, exposure to 1 M HCl and 1 M NaOH caused visible swelling, deformation, and partial loss of film integrity. This behavior is consistent with the known sensitivity of polysaccharide-based materials to strongly acidic and alkaline environments [58,59]. Hydrolysis of glycosidic linkages and disruption of physical associations may contribute to these changes, but these mechanisms were not directly characterized. The inability to recover coherent films after severe chemical exposure indicates irreversible macroscopic damage under the tested conditions [58].

Thermally induced repair was evaluated by comparing the mechanical properties of the original and repaired BC-BP films (Figure 9 and Figure 10; Table 7). The original film exhibited a tensile strength of 5.18 ± 0.21 MPa and a Young’s modulus of 0.471 ± 0.004 MPa. After repair, these values decreased to 2.85 ± 0.12 and 0.212 ± 0.001 MPa, respectively. These results correspond to recoveries of approximately 55% in tensile strength and 45% in Young’s modulus. Elongation at break remained nearly unchanged, decreasing only from 24.0 ± 1.0% to 23.7 ± 1.0%. Thus, the treatment partially restored strength and stiffness while largely preserving film extensibility. Reassociation of non-covalent interactions has been proposed as a repair mechanism in related biopolymer systems [28,40]. Hydrogen bonding and local polymer-chain rearrangement may therefore have contributed to the observed recovery [51,54]. However, the present mechanical and visual observations do not identify the molecular mechanism of repair. The incomplete recovery of tensile strength and Young’s modulus indicates that the original mechanical integrity was not fully restored. Overall, the BC-BP film demonstrated measurable but partial thermally induced repair under the applied treatment conditions.

Several limitations of the present study should be acknowledged. X-ray diffraction analysis was not performed; therefore, differences in crystallinity among bacterial cellulose, α-cellulose, and carboxylated cellulose nanofibers were not directly established, and the observed mechanical and thermal differences cannot be attributed specifically to cellulose crystallinity. TEM and SEM imaging of the composite films were also unavailable, preventing direct evaluation of filler dimensions, dispersion, aggregation, and interfacial organization within the starch matrix. Furthermore, the proposed hydrogen bonding, electrostatic associations, and chain entanglement were not directly validated by solid-state NMR, rheological analysis, SAXS, or DMA. The small differences in the FTIR O–H bands were not treated as quantitative evidence of hydrogen-bond-network formation because replicate FTIR measurements and an appropriate unreinforced reference spectrum were unavailable and the observed shifts were comparable to the spectral resolution. Similarly, TGA provides information on thermal degradation but cannot independently determine crystallinity, nanoscale dispersion, or intermolecular interactions. Optical characterization was limited to transmittance at 600 nm and therefore does not establish structural homogeneity, interfacial compatibility, or wavelength-dependent UV-barrier performance. In addition, water-vapor permeability, oxygen transmission, and other moisture-barrier properties were not measured; consequently, the suitability of these films for packaging applications remains preliminary. Future studies combining XRD, composite-film microscopy, TEM, solid-state structural analysis, full UV–Vis characterization, and quantitative gas- and moisture-barrier testing are required to validate the proposed structure–property relationships and packaging performance.

Future studies should systematically optimize the BC, Laponite, and glycerol contents to improve strength and stiffness without causing an excessive loss of extensibility. A controlled reduction in glycerol content, improved BC disintegration and distribution, and optimization of film-casting and drying conditions may enhance mechanical performance. The use of safe crosslinking strategies or suitable cellulose surface modification could also be evaluated. XRD, TEM or cross-sectional SEM, solid-state NMR, rheological analysis, and DMA will be needed to determine how these modifications affect crystallinity, filler dispersion, interfacial behavior, and mechanical properties. Water-vapor permeability, oxygen permeability, moisture-transmission, migration, and food-contact safety tests will also be required before the films can be considered suitable for packaging applications.

## 5. Conclusions

This study compared the thermal, optical, mechanical, solvent-exposure, and repair behavior of starch-based composite films containing BC, α-C, or CNC in the presence of Laponite and glycerol. The three cellulose-containing formulations exhibited different measured property profiles. Among the films containing different cellulose types, BC-BP showed the highest tensile strength and Young’s modulus. CNC-BP exhibited the highest elongation at break and the lowest thickness-normalized optical attenuation at 600 nm, whereas α-C-BP showed the highest T_max_ in the TGA analysis. Increasing the BC content from 0.25 to 1.0 g increased tensile strength from 3.17 ± 0.13 to 7.78 ± 0.31 MPa and Young’s modulus from 0.260 ± 0.002 to 0.996 ± 0.009 MPa. However, elongation at break decreased from 41 ± 1.6% to 16 ± 0.6%, demonstrating a trade-off between strength and extensibility. The maximum tensile strength remained within the lower range of values reported for several recently developed starch–cellulose composite films.

The BC-BP films retained their visible integrity following exposure to selected organic solvents but showed swelling, deformation, and partial loss of integrity under strongly acidic and alkaline conditions. Thermally induced repair restored approximately 55% of the original tensile strength and 45% of the original Young’s modulus while largely preserving elongation at break. These findings demonstrate measurable but incomplete mechanical recovery. Overall, cellulose type and BC content influenced the measured properties of the films. However, the present analyses do not establish the molecular or structural mechanisms responsible for these differences. Further investigations using XRD, TEM or cross-sectional SEM, solid-state NMR, rheological analysis, and DMA are required. Optimization of BC, Laponite, and glycerol contents may further improve mechanical performance. Barrier, migration, and food-contact safety tests will also be necessary before packaging suitability can be established.

## Figures and Tables

**Table 1:** Composition of starch–BC–Laponite bioplastic film formulations.

Formulation	Corn Starch (g)	Dry BC (g)	BC Suspension (2.94 wt.% Solids, g)	Distilled Water (mL)	Glycerol (99.5%, mL)	Laponite Suspension (1% *w*/*v*, mL)
Low (BC-0.25)	5.00	0.25	8.5	86.3	2.0	5.5
Mid (BC-0.50)	5.00	0.50	17.0	78.0	2.0	5.5
High (BC-1.00)	5.00	1.00	34.0	61.5	2.0	5.5

**Table 2:** Thermal degradation parameters of bioplastic films reinforced with different cellulose types.

Sample	T_onset_ (°C)	T_max_ (°C)	Residue at 600 °C (%)
α-C-BP	~270	~315	~18
CNC-BP	~260	~305	~16
BC-BP	~240	~295	~14

**Table 3:** Optical transparency and thickness of starch-based bioplastic films containing different cellulose components.

Sample	Transparency (mm^−1^)	Transmittance (%)	Thickness (mm)
CNC-BP	0.38 ± 0.09	75.5	0.33
α-C-BP	0.74 ± 0.26	59.8	0.31
BC-BP	0.84 ± 0.10	65.0	0.24

**Table 4:** Mechanical properties of starch-based bioplastic films reinforced with different cellulose types.

Sample	Thickness (mm)	Tensile Strength (MPa)	Elongation at Break (%)	Young’s Modulus (MPa)
BC-BP	0.24	4.47	52.0	0.229
CNC-BP	0.33	3.40	100.0	0.0456
α-C-BP	0.31	4.00	89.7	0.0611

**Table 5:** Mechanical properties of BC-reinforced bioplastic films with different BC contents.

Sample	Thickness (mm)	Tensile Strength (MPa)	Elongation at Break (%)	Young’s Modulus (MPa)
BC-BP 0.25	0.24	3.17 ± 0.13	41 ± 1.6	0.260 ± 0.002
BC-BP 0.5	0.25	5.18 ± 0.21	24 ± 1.0	0.471 ± 0.004
BC-BP 1.0	0.22	7.78 ± 0.31	16 ± 0.6	0.996 ± 0.009

Values are presented as mean ± standard deviation (SD) (*n* = 3).

**Table 6:** Swelling behavior of BC-BP bioplastic films in water at different immersion times.

Time (h)	Sample 1 (%)	Sample 2 (%)	Sample 3 (%)	Mean ± SD (%)
1	182.3	157.1	86.6	142.0 ± 48.7
3	183.9	171.8	102.5	152.7 ± 43.6
6	195.5	186.4	122.7	168.2 ± 39.8
24	212.6	207.6	127.0	182.4 ± 47.1

**Table 7:** Mechanical properties of the original and rehealed BC-BP bioplastic films.

Sample	Tensile Strength (MPa)	Elongation at Break (%)	Young’s Modulus (MPa)
Original BC-BP	5.18 ± 0.21	24.0 ± 1.0	0.471 ± 0.004
Rehealed BC-BP	2.85 ± 0.12	23.7 ± 1.0	0.212 ± 0.001

Values are presented as mean ± standard deviation (SD) (*n* = 3).

**Table 8:** Comparison of the mechanical properties of the developed BC-reinforced starch bioplastics with previously reported starch-, cellulose-, and clay-based composite films.

Material/Formulation	Cellulose Reinforcement	Plasticizer/Additives	Thickness (mm)	Tensile Strength (MPa)	Elongation at Break (%)	Young’s Modulus (MPa)	Test Method	Ref.
BC-BP 0.25 (present study)	BC, 0.25 g dry basis	Glycerol/Laponite	0.24	3.17 ± 0.13	41 ± 1.6	0.260 ± 0.002	Adapted ASTM D882	This study
BC-BP 0.5 (present study)	BC, 0.50 g dry basis	Glycerol/Laponite	0.25	5.18 ± 0.21	24 ± 1.0	0.471 ± 0.004	Adapted ASTM D882	This study
BC-BP 1.0 (present study)	BC, 1.00 g dry basis	Glycerol/Laponite	0.22	7.78 ± 0.31	16 ± 0.6	0.996 ± 0.009	Adapted ASTM D882	This study
Corn-starch/CNF film (NCO-2)	CNF, 4.16 g	Glycerol, 4 g	0.0922 ± 0.0074	25.58 ± 3.05	2.76 ± 0.21	1728 ± 181.9	ASTM D882-09; 24 × 150 mm; gauge length 100 mm; 9 mm/min	[20]
Corn-starch/CNP/nanoclay film (1.5/0.3-CNPs/NC)	CNP, 1.5 wt.%	Glycerol, 30 wt.%; nanoclay, 0.3 wt.%	NR	4.885	14.8 ^1^	NR ^2^	ASTM D3039-76; five specimens	[21]
Huaya-starch/bentonite film (Starch-5) ^3^	None	Glycerol, 1.6 g; bentonite clay, 5 wt.%	0.31 ± 0.03	5.24 ± 0.40	7.53 ± 1.43	338.62 ± 75.70	ASTM D882; 25 × 100 mm; gauge length 50 mm; 50 mm/min; 25 °C and 50% RH	[22]
Commelina-starch/CNC/CNF film (CNC1-CNF1)	CNC, 1 wt.%; CNF, 1 wt.%	Glycerol, 30% of dry starch	0.10 ± 0.02	39.9 ± 6.0	1.9 ± 0.4	2530 ± 45	Lloyd TAPlus; 10 × 100 mm; gauge length 40 mm; 60 mm/min; 57% RH for 24 h; *n* ≥ 3	[23]
Corn-starch/nanocellulose film	Nanocellulose, 6 wt.%	Glycerol, 1.5 g per 5 g starch	NR	10.54 ± 0.87	12.51 ± 1.60	NR	ASTM D882-18; 10 mm/min; 23 °C and 50% RH for ≥48 h; *n* = 3	[24]
Cassava-starch/lignin/CNF film, optimized formulation	CNF, 5.00 wt.%	Glycerol, 50 wt.%; lignin, 4.81 wt.%	Approximately 0.12	21.51	48.81	25.76	ASTM D882; 1.3 mm/min; 25 °C and 55% RH; *n* = 3	[25]
Corn-starch/unbleached CNF film (6.0 wt.% ubCNF)	Unbleached CNF, 6.0 wt.% relative to starch	Glycerol, 30 wt.% relative to starch	0.223 ± 0.008	10.16	NR ^4^	277.3	ASTM D882-18; specimens 10 × 170 mm; 25 mm/min; conditioned at 50% RH for 48 h	[26]
Cassava-starch/MCC film (Starch/5%MCC)	MCC, 5 wt.% relative to starch (0.25 g MCC per 5 g starch)	Glycerol, 1.5 g per 5 g starch	0.144 ± 0.009	11.18 ± 1.46	4.15 ± 0.18	488.89 ± 105.6	TA.XT-PLUS texture analyzer; specimen 50 × 10 mm; gauge length 30 mm; 10 mm/min; *n* = 5 ^5^	[27]
Potato-starch/TEMPO-oxidized cellulose film (cGAP_TEMPO-st)	TEMPO-oxidized cellulose fibers from gloss art-paper waste; cellulose-to-starch ratio 1:1	25 wt.% glycerol aqueous solution	0.10 ^6^	71.32	Up to 13	NR ^7^	IMC-18E0 tensile tester; dumbbell specimen 65 × 12 × 0.10 mm; narrow Section 4 mm; gauge length 20 mm; 5 mm/min; 23 °C; *n* = 5	[28]
Cassava-starch/CNF/nano-SiO_2_ film, optimized formulation	CNF, 1.38% (*w*/*v*)	Glycerol, 0.7 wt.% relative to starch; SiO_2_ nanoparticles, 0.30% (*w*/*v*)	0.087 ± 0.014	5.813	12.3654	NR	Universal testing machine; specimens 20 × 100 mm; initial grip separation 70 mm; 2 mm/s; conditioned at 25 ± 1 °C and 50 ± 2% RH for 24 h; *n* = 5 ^8^	[29]
Corn-starch/black-seed/cassava-bagasse-fiber film (CS-BS/CB9%)	Cassava bagasse fibers, 9 wt.% relative to dry starch	Fructose and glycerol, 30 wt.% relative to dry starch; black seed, 9 wt.%	NR ^9^	18.22	10.85	118.32	ASTM D882 (2002); INSTRON 5-kN tensile machine; specimens 10 × 70 mm; 2 mm/min; 30 °C; n = 5	[30]
Tapioca-starch/chitosan/BC-nanofiber film (GU/CH/20BC)	Disintegrated bacterial cellulose nanofibers, 0.136 g	Glycerol, 2 mL; chitosan, 2.5 g; acetic acid solution, 100 mL	NR	4.7	21.5	NR	ASTM D638 Type V; COM-TEN 95T Series 5K; conditioned at 25 °C and 50 ± 5% RH for 48 h; tested at room temperature and 75% RH; 5 mm/min; *n* ≥ 3	[31]

Values are reported as presented in the original studies. NR, not reported; BC, bacterial cellulose; CNF, cellulose nanofibrils; CNC, cellulose nanocrystals; CNP, cellulose nanoparticles; MCC, microcrystalline cellulose; ubCNF, unbleached cellulose nanofibrils; RH, relative humidity. Direct comparison should be made cautiously because the formulations, specimen dimensions, film thicknesses, conditioning procedures, crosshead speeds, and testing standards differed among studies. ^1^ Converted from the reported strain at failure of 0.148 mm/mm. ^2^ Reference [21] reports a tensile modulus of 142.945 GPa. This value was excluded because the reported unit appears internally inconsistent and requires verification. ^3^ Reference [22] contains bentonite clay without cellulose and is included as a starch–clay comparator relevant to the Laponite-containing films. ^4^ In Reference [26], elongation at break was presented graphically without an exact numerical value and was therefore recorded as NR. ^5^ Reference [27] did not specify an ASTM or ISO standard for tensile testing; the reported instrument and testing conditions are provided instead. ^6^ Reference [28] reports the thickness of the molded tensile specimen rather than the mean film thickness. ^7^ In Reference [28], Young’s modulus was presented graphically without an exact numerical value and was therefore recorded as NR. ^8^ Reference [29] did not specify an ASTM or ISO standard for tensile testing; the reported instrument and testing conditions are provided instead. Young’s modulus was not reported. ^9^ Reference [30] reports a thickness of 0.35 ± 0.04 µm for CS-BS/CB9%. Because this value and unit appear internally inconsistent for a cast starch film, the thickness was recorded as NR and was not converted to millimeters.

## Data Availability

The raw data supporting the conclusions of this article will be made available by the authors on request.

## Supplementary Materials

The following supporting information can be downloaded at: https://www.mdpi.com/article/10.3390/polym18182190/s1, Figure S1: Zeta potential of a 1 wt% aqueous Laponite dispersion; Figure S2: SEM images of cellulose matrices: (a) α-C at 500×, (b) α-C at 2000×, (c) CNC at 1000×, and (d) CNC at 3000×; Table S1: Determination of Water and Solid Content of BC.

## Abbreviations

The following abbreviations are used in this manuscript:

BC	Bacterial cellulose
CNC	Carboxylated cellulose nanofibers
α-C	α-Cellulose
BP	Bioplastic
BC-BP	Bacterial cellulose-reinforced bioplastic
CNC-BP	Carboxylated cellulose nanofiber-reinforced bioplastic
α-C-BP	α-Cellulose-reinforced bioplastic
FTIR	Fourier transform infrared
TGA	Thermogravimetric analysis
DTG	Derivative thermogravimetry
DLS	Dynamic light scattering
SEM	Scanning electron microscopy
LV-SE	Low-vacuum secondary electron
BSE	Backscattered electron
THF	Tetrahydrofuran
SD	Standard deviation
UV–Vis	Ultraviolet–visible
T_600_	Transmittance at 600 nm
T_max_	Temperature at maximum degradation rate
T_onset	Onset degradation temperature

## References

[1] Zhu Y., Romain C., Williams C.K. (2016). Sustainable Polymers from Renewable Resources. Nature.

[2] Liu C., Luan P., Li Q., Cheng Z., Sun X., Cao D., Zhu H. (2020). Biodegradable, Hygienic, and Compostable Tableware from Hybrid Sugarcane and Bamboo Fibers as Plastic Alternative. Matter.

[3] Gamage A., Thiviya P., Mani S., Ponnusamy P.G., Manamperi A., Evon P., Merah O., Madhujith T. (2022). Environmental Properties and Applications of Biodegradable Starch-Based Nanocomposites. Polymers.

[4] Kour M., Chaudhary S., Kumar R. (2025). Cellulose Based Bioplastics: A Sustainable Approach toward Environmental Safety—A Review. Sustain. Mater. Technol..

[5] Mose B.R., Maranga S.M. (2011). A Review on Starch Based Nanocomposites for Bioplastic Materials. J. Mater. Sci. Eng..

[6] Hossain M., Shahid M.A., Akter S., Ferdous J., Afroz K., Refat K., Faruk O., Jamal M., Uddin M.N., Samad M. (2024). Cellulose and Starch-Based Bioplastics: A Review of Advances and Challenges for Sustainability. Polym.-Plast. Technol. Mater..

[7] Zoungranan Y., Lynda E., Dobi-Brice K.K., Tchirioua E., Bakary C., Yannick D.D. (2020). Influence of Natural Factors on the Biodegradation of Simple and Composite Bioplastics Based on Cassava Starch and Corn Starch. J. Environ. Chem. Eng..

[8] Nandiyanto A.B.D., Fiandini M., Ragadhita R., Sukmafitri A., Salam H., Triawan F. (2020). Mechanical and Biodegradation Properties of Cornstarch-Based Bioplastic Material. Mater. Phys. Mech..

[9] Gurunathan M.K., Navasingh R.J.H., Selvam J.D.R., Čep R. (2025). Development and Characterization of Starch Bioplastics as a Sustainable Alternative for Packaging. Sci. Rep..

[10] Reichert C.L., Bugnicourt E., Coltelli M.B., Cinelli P., Lazzeri A., Canesi I., Braca F., Martínez B.M., Alonso R., Agostinis L. (2020). Bio-Based Packaging: Materials, Modifications, Industrial Applications and Sustainability. Polymers.

[11] Kim J., Choi W., Park H., Jo S., Park K., Cho H., Oh Y., Choi M., Choi B., Ryu D.Y. (2025). Tunable Mechanical Properties in Biodegradable Cellulosic Bioplastics Achieved via Ring-Opening Polymerization. ACS Nano.

[12] Oliveira R.L., Barud H.S., De Salvi D.T.B., Perotti G.F., Ribeiro S.J.L., Constantino V.R.L. (2015). Transparent Organic-Inorganic Nanocomposite Membranes Based on Carboxymethylcellulose and Synthetic Clay. Ind. Crops Prod..

[13] Azeredo H.M.C., Barud H., Farinas C.S., Vasconcellos V.M., Claro A.M. (2019). Bacterial Cellulose as a Raw Material for Food and Food Packaging Applications. Front. Sustain. Food Syst..

[14] Gullo M., Sola A., Zanichelli G., Montorsi M., Messori M., Giudici P. (2017). Increased Production of Bacterial Cellulose as Starting Point for Scaled-Up Applications. Appl. Microbiol. Biotechnol..

[15] Yuan Z., Fan Q., Dai X., Zhao C., Lv A., Zhang J., Xu G., Qin M. (2014). Cross-Linkage Effect of Cellulose/Laponite Hybrids in Aqueous Dispersions and Solid Films. Carbohydr. Polym..

[16] Valencia G.A., Luciano C.G., Lourenço R.V., do Amaral Sobral P.J. (2018). Microstructure and Physical Properties of Nano-Biocomposite Films Based on Cassava Starch and Laponite. Int. J. Biol. Macromol..

[17] (2018). Standard Test Method for Tensile Properties of Thin Plastic Sheeting.

[18] Gea S., Pasaribu K.M., Sarumaha A.A., Rahayu S. (2022). Cassava Starch/Bacterial Cellulose-Based Bioplastics with *Zanthoxylum acanthopodium*. Biodivers. J. Biol. Divers..

[19] Cazón P., Vázquez M. (2021). Bacterial Cellulose as a Biodegradable Food Packaging Material: A Review. Food Hydrocoll..

[20] Garuti G.L., de Freitas R.R.M., de Lima V.H., do Carmo K.P., de Pádua F.A., Botaro V.R. (2024). Nanocellulose reinforced starch biocomposite films via tape-casting technique. Polímeros.

[21] Olusanya J.O., Mohan T.P., Kanny K. (2023). Effect of nanoclay (NC) on mechanical and thermal properties of cellulose nanoparticles (CNPs) filled cornstarch bioplastic film. J. Polym. Res..

[22] Pech-Cohuo S.C., Dzul-Cervantes M.A.d.A., Pérez-Pacheco E., Canto Rosado J.A., Chim-Chi Y.A., Ríos-Soberanis C.R., Cuevas-Carballo Z.B., Uc-Cayetano E.G., Can-Herrera L.A., Ortíz-Fernández A. (2024). Effect of clays incorporation on properties of thermoplastic starch/clay composite bio-based polymer blends. Sci. Rep..

[23] García-Guzmán L., Velazquez G., Arzate-Vázquez I., Castaño-Rivera P., Guerra-Valle M., Castaño J., Guadarrama-Lezama A.Y. (2024). Preparation of nanocomposite biopolymer films from *Commelina coelestis* Willd starch and their nanostructures as a potential replacement for single-use polymers. Foods.

[24] Malekzadeh E., Tatari A., Dehghani Firouzabadi M. (2024). Effects of biodegradation of starch-nanocellulose films incorporated with black tea extract on soil quality. Sci. Rep..

[25] AbdulRasheed-Adeleke T., Egwim E.C., Sadiku E.R., Ochigbo S.S. (2022). Optimization of lignin-cellulose nanofiber-filled thermoplastic starch composite film production for potential application in food packaging. Molecules.

[26] Lomelí-Ramírez M.G., Reyes-Alfaro B., Martínez-Salcedo S.L., González-Pérez M.M., Gallardo-Sánchez M.A., Landázuri-Gómez G., Vargas-Radillo J.J., Diaz-Vidal T., Torres-Rendón J.G., Macias-Balleza E.R. (2023). Thermoplastic starch biocomposite films reinforced with nanocellulose from *Agave tequilana* Weber var. Azul bagasse. Polymers.

[27] Jeencham R., Chiaoketwit N., Numpaisal P.-o., Ruksakulpiwat Y. (2024). Study of biocomposite films based on cassava starch and microcrystalline cellulose derived from cassava pulp for potential medical packaging applications. Appl. Sci..

[28] Hafid H.S., Omar F.N., Bahrin E.K., Wakisaka M. (2023). Extraction and surface modification of cellulose fibers and its reinforcement in starch-based film for packaging composites. Bioresour. Bioprocess..

[29] Bie M., Wang T., Yang Z., Yuan S., Gu Y., Liu C., Zhao W., Song K. (2025). Metaheuristic-optimized cassava starch/CNF/SiO_2_ bio-nanocomposite films for sustainable food packaging: A data-driven approach. Sustainability.

[30] Abotbina W., Sapuan S.M., Ilyas R.A., Sultan M.T.H., Alkbir M.F.M. (2022). Preparation and characterization of black seed/cassava bagasse fiber-reinforced cornstarch-based hybrid composites. Sustainability.

[31] Abral H., Pratama A.B., Handayani D., Mahardika M., Aminah I., Sandrawati N., Sugiarti E., Muslimin A.N., Sapuan S.M., Ilyas R.A. (2021). Antimicrobial edible film prepared from bacterial cellulose nanofibers/starch/chitosan for a food packaging alternative. Int. J. Polym. Sci..

[32] Jayarathna S., Andersson M., Andersson R. (2022). Recent Advances in Starch-Based Blends and Composites for Bioplastics Applications. Polymers.

[33] Bartmański M., Pawłowski Ł., Zieliński A., Mielewczyk-Gryń A., Strugała G., Cieślik B. (2020). Electrophoretic Deposition and Characteristics of Chitosan–Nanosilver Composite Coatings on a Nanotubular TiO_2_ Layer. Coatings.

[34] Garcia-Padilla E., Morales-Sanchez M.A., Martinez-Bustos M.E. (2018). Synthesis and Characterization of Starch/Na-MMT Nanocomposites. Contemp. Eng. Sci..

[35] Swain S.K., Budhraja P.P., Behera L., Cho H. (2014). Study of Thermal, Oxygen-Barrier, Fire-Retardant and Biodegradable Properties of Starch Bionanocomposites. Polym. Compos..

[36] Hussain M., Ullah S., Aziz M., Raza A. (2018). Hybrid Monolith of Graphene/TEMPO-Oxidized Cellulose Nanofiber as Mechanically Robust, Highly Functional, and Recyclable Adsorbent of Methylene Blue Dye. J. Nanomater..

[37] Zhong Z., Sun Y., Zheng J., Li J., Liu K. (2022). Evaluation of Aerogel Spheres Derived from *Salix psammophila* in Removal of Heavy Metal Ions in Aqueous Solution. Forests.

[38] Morcillo-Martín N., Rodríguez A., Gandini A. (2022). Cellulose Nanofiber-Based Aerogels from Wheat Straw: Influence of Surface Load and Lignin Content on Their Properties and Dye Removal Capacity. Biomolecules.

[39] Yu Y., Wang X., Liu Y., Xu X. (2023). Structure, Rheological Properties, and Biocompatibility of Laponite® Cross-Linked Starch/Polyvinyl Alcohol Hydrogels. Int. J. Biol. Macromol..

[40] Luo D., Sun G., Wang Y., Shu X., Chen J., Sun M., Liu X., Liu C., Xiao H., Xu T. (2024). Metal Ion and Hydrogen Bonding Synergistically Mediated Carboxylated Lignin/Cellulose Nanofibrils Composite Film. Carbohydr. Polym..

[41] Nascimento D.M., Nunes Y.L., Figueirêdo M.E., Azeredo H.N., Aouada C.M., Feitosa J.R., Rosa M.F., Dufresne A. (2016). Nanocellulose Produced from Rice Hulls and Its Effect on the Properties of Biodegradable Starch Films. Mater. Res..

[42] Csiszár E., Fekete P., Tóth A., Bozó E., Koczka A., Miskolczi B. (2021). The Role of Structure and Interactions in Thermoplastic Starch-Nanocellulose Composites. Polymers.

[43] Guzman-Puyol S., Heredia-Guerrero J.A., Ceseracciu L., Hajiali P., Canale R., Scarpellini L., Cingolani G., Bayer I.S., Athanassiou A., Benítez J.J. (2019). Transparent and Robust All-Cellulose Nanocomposite Packaging Materials Prepared in a Mixture of Trifluoroacetic Acid and Trifluoroacetic Anhydride. Nanomaterials.

[44] Hietala M., Mathew A.P., Oksman K. (2013). Bionanocomposites of Thermoplastic Starch and Cellulose Nanofibers Manufactured Using Twin-Screw Extrusion. Eur. Polym. J..

[45] Faria M. (2015). New Bacterial Cellulose Nanocomposites Prepared by In Situ Radical Polymerization of Methacrylate Monomers. Master’s Thesis.

[46] Ahmed J., Tiwari B.K., Imam S.H., Rao M.A. (2021). Microbial Cellulose Based Films and Composites for Food Packaging: A Review. Food Technol..

[47] Perotti G.J., Troiani H.A., Zampieri M., Errea M.I., Morini N.J. (2013). Biopolymer-Clay Nanocomposites: Cassava Starch and Synthetic Clay Cast Films. J. Braz. Chem. Soc..

[48] Humairah N., Mariatti M., Kamarudin M. (2012). Properties of Sago Starch-Nanoclay Biocomposites Film. Adv. Mater. Res..

[49] Silva S.P., Sabino M.A., Fernandes E.M., Correlo V.M., Boesel L.F., Reis R.L. (2012). A Fundamental Investigation of the Microarchitecture and Mechanical Properties of TEMPO-Oxidized Nanofibrillated Cellulose (NFC)-Based Aerogels. Cellulose.

[50] Fekete E., Pukánszky B., Tóth A., Bertóti I. (2022). The Effect of Surface Characteristics of Clays on the Properties of Starch Nanocomposites. Materials.

[51] Qi G., Li N., Wang D., Sun X.S. (2016). Development of High-Strength Soy Protein Adhesives Modified with Sodium Montmorillonite Clay. J. Am. Oil Chem. Soc..

[52] Gabr M.H., Okumura W., Ueda H., Kuriyama W., Uzawa I., Kimpara T. (2013). Mechanical, Thermal, and Moisture Absorption Properties of Nano-Clay Reinforced Nano-Cellulose Biocomposites. Cellulose.

[53] Wang Y., Chen X., Dufresne A. (2015). Cellulose/Montmorillonite Nanocomposite Films: Preparation and Properties. Cellulose.

[54] Guo G., Tian H., Wu Q. (2019). Influence of pH on the Structure and Properties of Soy Protein/Montmorillonite Nanocomposite Prepared by Aqueous Solution Intercalating. Appl. Clay Sci..

[55] Chen B., Evans J.R.G. (2006). Highly Exfoliated Soy Protein/Montmorillonite Nanocomposites. Biomacromolecules.

[56] Introzzi L., Fuentes-Alventosa J.M., Cozzolino C.A., Trabattoni S., Tavazzi S., Bianchi C.L., Schiraldi A., Piergiovanni L., Farris S. (2012). Ultrasound-Assisted Pullulan/Montmorillonite Bionanocomposite Coating with High Oxygen Barrier Properties. Langmuir.

[57] Sharma R., Jafari S., Sharma S. (2017). Biodegradable Starch/PVOH/Laponite RD-Based Bionanocomposite Films Coated with Graphene Oxide: Preparation and Performance Characterization for Food Packaging Applications. Colloid Polym. Sci..

[58] Silva S.S., Oliveira S.M., Oliveira A., Reis R.L. (2012). Application of Infrared Spectroscopy to Analysis of Chitosan/Clay Nanocomposites. Infrared Spectroscopy—Materials Science, Engineering and Technology.

[59] Paluszkiewicz C., Stodolak M., Hasik M., Błażewicz M. (2011). FT-IR Study of Montmorillonite–Chitosan Nanocomposite Materials. Spectrochim. Acta A Mol. Biomol. Spectrosc..

