# Supplementary material for: Bacterial Cellulose- and Laponite-Reinforced Corn Starch Bioplastics for Sustainable Packaging
Source: Polymers (Basel). 2026 Sep 8;18(18):2190. doi: 10.3390/polym18182190 (PMC13611537; doi:10.3390/polym18182190)
Supplement: Supplementary file 1 [file polymers-18-02190-s001.zip › polymers-4507126-supplementary.pdf]

Development and Characterization of Corn Starch-Based Bioplastics Reinforced with Bacterial Cellulose and Laponite for Sustainable Packaging Application

Rysgul Tuleyeva [1,2], Nargiz Gizatullina [1], Alexey Shakhvorostov [3], Zhanserik Shynykul [1,4,*], Gaukhar Toleutay [1,5,*]

[1] Department of Chemical and Biochemical Engineering, Geology and Oil-Gas Business Institute Named After K. Turyssov, Satbayev University, Almaty 050043, Kazakhstan
[2] Department of Chemistry and Technology of Organic Substances, Natural Compounds and Polymers, Faculty of Chemistry and Chemical Technology, Al-Farabi Kazakh National University, Almaty 050040, Kazakhstan
[3] Institute of Polymer Materials and Technology, Almaty 050019, Kazakhstan
[4] Department of Pharmaceutical Sciences, College of Pharmacy, The University of Tennessee, Health Science Center, Memphis, TN 38103, USA
[5] Department of Chemistry, University of Tennessee, Knoxville, TN 37996, USA
*e-mail: zshynyku@uthsc.edu (Z.S.), gaukhar.toleutay@gmail.com (G.T.)

Determination of Water and Solid Content of BC

The results of moisture determination for the commercial bacterial cellulose are presented in Table S1. The obtained values indicate that the material contained a very high proportion of water, typical for hydrogel-type bacterial cellulose.

Table S1 - Determination of Water and Solid Content of BC

Sample	Wet mass (g)	Dry mass (g)	Water content (%)	Solid content (%)
1	1.0060	0.0256	97.46	2.54
2	1.1906	0.0376	96.84	3.16
3	1.0737	0.0335	96.88	3.12
Mean ± SD	—	—	**97.06 ± 0.34**	**2.94 ± 0.34**

The average water content of the commercial BC was 97.06 ± 0.34 % (SD), corresponding to a solid fraction of approximately 2.94 ± 0.34 %. The standard error of the mean (SEM) was ± 0.20 %. Such a high water content is characteristic of the nanofibrillar hydrogel network of bacterial cellulose, in which water is strongly retained through hydrogen bonding within the three-dimensional structure.

The obtained results are consistent with previously reported data for commercial and laboratory-produced BC, which typically contain 96-99 % water depending on the

degree of purification and drying method. The gravimetric drying at 105°C effectively removes both free and loosely bound water without causing noticeable thermal degradation of cellulose.

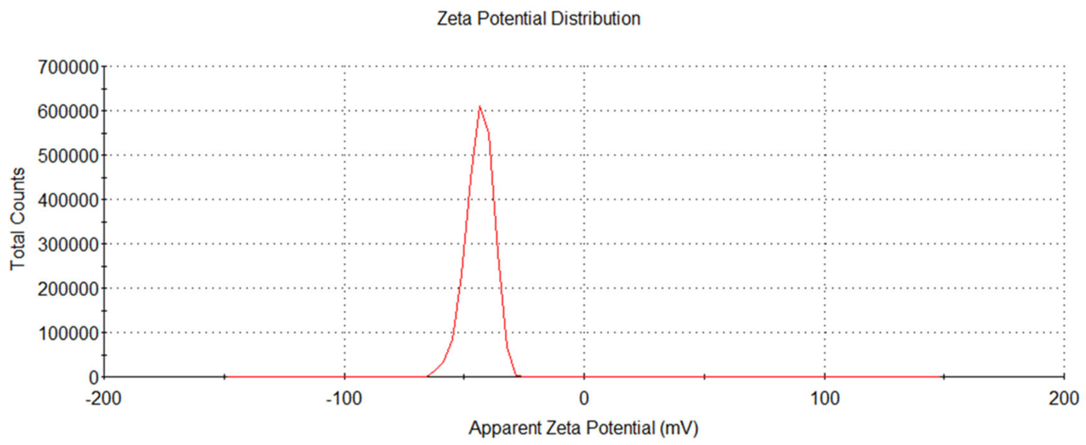

Figure S1. Zeta potential of a 1 wt% aqueous Laponite dispersion.

The ζ-potential of a 1wt% aqueous Laponite dispersion was measured at 25 °C using the Smoluchowski model. The mean ζ-potential was -43.2 ± 3.8 mV (n = 3), with an electrophoretic mobility of -3.39 ± 0.29 µm·cm/V·s and a conductivity of 0.49 ± 0.01 mS/cm, indicating good colloidal stability of the dispersion.

SEM analysis further confirmed the morphological differences between the two cellulose types (Figure S2). The α-C exhibited large, block-like particles with smooth surfaces, typical for microcrystalline cellulose. In contrast, CNC showed a highly entangled fibrillar network, forming a porous, web-like structure. This morphology provides a higher interfacial area, which is advantageous for composite formation and is consistent with the enhanced thermal stability observed in TGA analysis.

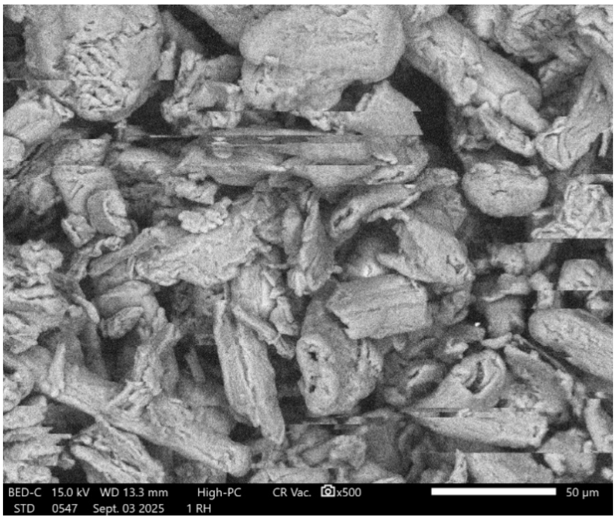

A)

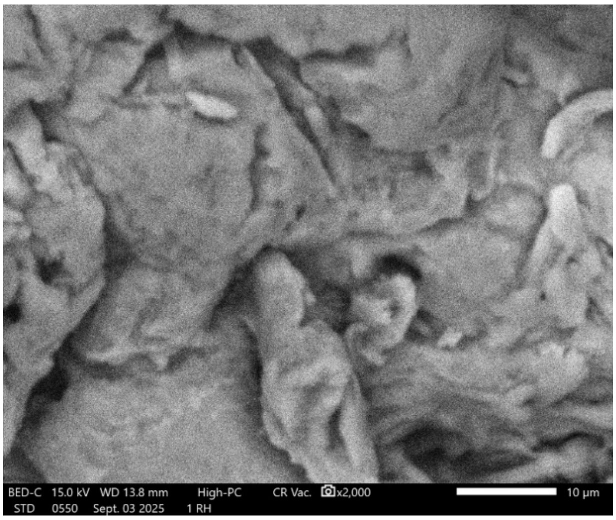

B)

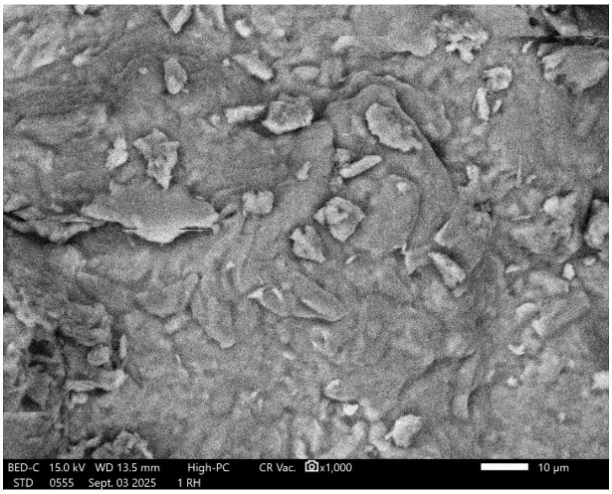

C)

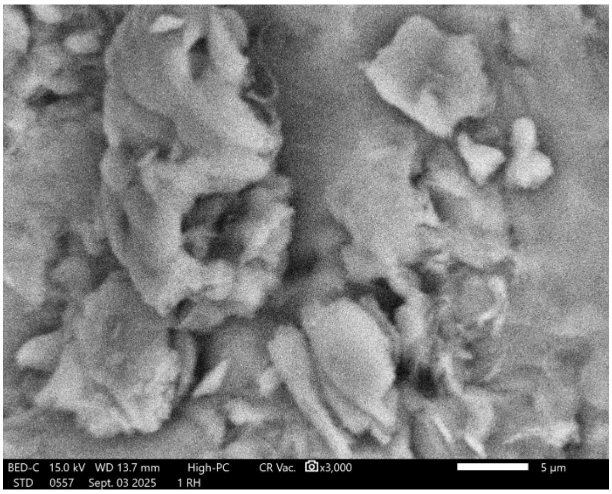

D)

Figure S2. SEM images of cellulose matrices: (a) α-C at 500×, (b) α-C at 2000×, (c) CNC at 1000×, and (d) CNC at 3000×.
